# Generative AI Mental Health Chatbots as Therapeutic Tools: Systematic Review and Meta-Analysis of Their Role in Reducing Mental Health Issues

**DOI:** 10.2196/78238

**Published:** 2025-12-16

**Authors:** Qiyang Zhang, Renwen Zhang, Yiying Xiong, Yuan Sui, Chang Tong, Fu-Hung Lin

**Affiliations:** 1 Department of Educational Advancement, Duke-NUS Medical School, 8 College Road, Singapore, 169857, Singapore, 65 66012186; 2 Wee Kim Wee School of Communication and Information, Nanyang Technological University, Singapore, Singapore; 3 School of Education, Johns Hopkins University, Baltimore, MD, United States

**Keywords:** generative artificial intelligence, AI chatbot, conversational agents, mental health, meta-analysis, systematic review, artificial intelligence

## Abstract

**Background:**

In recent years, artificial intelligence (AI) has driven the rapid development of AI mental health chatbots. Most current reviews investigated the effectiveness of rule-based or retrieval-based chatbots. To date, there is no comprehensive review that systematically synthesizes the effect of generative AI (GenAI) chatbot’s impact on mental health.

**Objective:**

This review aims to (1) narratively synthesize existing GenAI mental health chatbots’ technical features, treatment and research designs, and sample characteristics through a systematic review of quantitative studies and (2) quantify the effectiveness and key moderators of these rigorously designed trials on GenAI mental health chatbots through a meta-analysis of only randomized controlled trials (RCTs).

**Methods:**

The search strategy includes 11 database searching, backward citation tracking, and a manual ad hoc search to update literature. This thorough literature search, completed in March 2025, returned 5555 records for screening. The systematic review included studies that (1) used generative or hybrid (rule/retrieval-based and generative) AI-based chatbots to deliver interventions and (2) quantitatively measured mental health-related outcomes. The meta-analysis has additional inclusion criteria: (1) studies must be RCTs, (2) must measure negative mental health issues, (3) the comparison group must not have chatbot features, and (4) must provide enough statistics for effect size calculation. We followed the PRISMA (Preferred Reporting Items for Systematic Reviews and Meta-Analyses) checklist and registered the protocol retrospectively during the revision process (September 18, 2025). In meta-regression, data were synthesized in R software using a random-effects model.

**Results:**

The narrative synthesis of 26 studies revealed that (1) GenAI chatbot interventions mostly took place in non-WEIRD countries (non-Western, Educated, Industrialized, Rich, and Democratic) and (2) there is a lack of studies focusing on young children and older adults. The meta-analysis of 14 RCTs showed a statistically significant effect (effect size [ES]=0.30, *P*=.047, N=6314, 95% CI 0.004, 0.59, 95% prediction interval [PI] −0.85, 1.67), which means that GenAI chatbots are, on average, effective in reducing negative mental health issues, such as depression, anxiety, among others. We found that social-oriented chatbots (ie, those that mainly provide social interactions) are more effective than task-oriented programs (ie, those that assist with specific tasks). Risk of bias in the nonrandomized studies and RCTs was assessed using Cochrane ROBINS-I (Risk Of Bias In Non-randomised Studies – of Interventions) and RoB2 (revised Cochrane risk-of-bias tool for randomized trials), respectively, indicating a moderate amount of risk. One main limitation of this meta-analysis is the small number of studies (n=14) included.

**Conclusions:**

By identifying research gaps, we suggest that future researchers investigate user groups such as adolescents and older adults, outcomes other than depression and anxiety, cultural adaptations in non-WEIRD countries, ways to streamline chatbots in usual care practices, and explore applications in diverse settings. More importantly, we cannot ignore GenAI chatbots’ risks while acknowledging their promise. This review also emphasized several ethical implications.

## Introduction

### Background of Generative Artificial Intelligence (GenAI) Mental Health Chatbots

Globally, one in every eight individuals is affected by a mental health issue [1], which can significantly impair people’s physical health [2] and wellness [3]. Mental health conditions impose an estimated $1.9 trillion [4] economic burden worldwide. Despite the high social cost and pressing need for treatment, access to mental health services remains severely limited [5]. In fact, over 70% of people with mental disorders receive no treatment from professionals due to stigma, counselors’ shortage, and under-resourced care infrastructures [6]. The COVID-19 pandemic has further exacerbated these challenges, highlighting the urgent need for scalable, accessible, and cost-effective interventions [7].

Recent advances in artificial intelligence (AI) have driven the rapid development of mental health chatbots [8,9]. These AI chatbot-based interventions offer 24/7 support, enhanced self-management [10], reduced stigma, and appeal to digital-native users [11]. Multimedia Appendix 1 presents an overview of existing AI mental health chatbots. A number of reviews showed promising effects of AI chatbot interventions on reducing mental health distress and improving quality of life [10,12-16] (see Multimedia Appendix 2). However, most of these interventions relied on retrieval-based chatbots, which use predefined responses or static databases and often result in rigid, repetitive interactions [17-19].

In contrast, GenAI chatbots, powered by large language models (LLMs), such as GPT models, generate novel responses in real time. By tailoring replies to the user’s language, tone, and emotional content, they enable conversations that are more natural, personalized, and emotionally resonant. This capacity may strengthen user engagement [20], therapeutic alliance [21], and the sense of being understood [22]. A recent meta-analysis shows that GenAI chatbots outperform rule-based and retrieval-based chatbots in reducing depressive symptoms [13]. Emerging primary studies suggest that GenAI mental health chatbots can improve engagement and adherence by supporting between-session cognitive behavioral therapy tasks [23] and delivering positive psychology interventions, such as gratitude or self-reflection exercises [24]. Beyond structured therapy, companion chatbots such as Replika have been found to reduce loneliness [25] and produce outcomes comparable to mindfulness interventions among older adults [26]. Despite this huge potential, no review currently exists that has systematically synthesized the effect of GenAI chatbots’ impact on mental health.

### Aims of This Review

This review seeks to fill this gap by conducting a systematic review and meta-analysis of GenAI chatbot interventions for mental health. This paper aims to:

1. synthesize current GenAI mental health chatbots’ technical and treatment features, research designs, and sample characteristics,
2. quantify the effectiveness of these interventions via a meta-analysis of randomized controlled trials (RCTs)
3. examine key moderators of intervention effectiveness, including chatbot design, population characteristics, intervention context, and outcome types.

## Methods

### Search Strategy

To ensure comprehensive coverage of the literature, the first author (QZ) implemented a thorough search strategy that included database queries, updated manual searches, and backward citation tracking. Using a predefined set of keywords, the author systematically searched 11 databases, including Scopus, Embase, Web of Science, APA PsycInfo, Child Development & Adolescent Studies, ERIC, ACM Digital Library, CINAHL, MEDLINE, PsyArXiv, and OpenDissertations. We developed a predefined set of keywords on “method,” “generative AI chatbot,” and “mental health” (keywords detailed in Multimedia Appendix 3 and search details can be found in Multimedia Appendix 4). Furthermore, through a tool called *CitationChaser*, we conducted backward citation tracking for 9 similar reviews listed in Multimedia Appendix 2. The data search was completed on November 1, 2024. In order to update our search, on March 5, 2025, we conducted another round of manual ad hoc search to seek newly published studies. Together, 5555 records were identified from the combined search strategies.

### Inclusion Criteria for Systematic Review

1. Studies should have used generative or hybrid (rule or retrieval-based and generative) AI-based conversational agent or chatbot to deliver interventions. For example, rule-based chatbots that formulate responses to user queries through a predetermined set of rules without employing any AI algorithms or techniques were excluded (eg [27]).
2. Studies must quantitatively measure mental health-related outcomes, including both positive and negative constructs.
3. These must be primary studies. We excluded review papers.
4. Text must be available on the internet or written in English.
5. Studies must be published on or after January 1, 2014, as the modern generative AI chatbot era began around 2015 with neural conversational models [28].

### Inclusion Criteria for Meta-Analysis

Apart from the above criteria, eligible studies for meta-analysis must meet additional inclusion criteria:

1. The study must quantitatively measure negative mental health issues in the outcome, such as depression, anxiety, psychological distress, stress, etc. We excluded well-being, happiness, positive emotions, etc. due to the scope of this study. The study by Vowels et al [29] was excluded because of its focus on positive well-being only. We also excluded usability studies that measure outcomes using scales such as System Usability Scale.
2. Studies must be RCTs. We excluded single-arm studies. The study by Zheng [30] was excluded because the study design is not RCT.
3. The comparison group must not have chatbot features since the treatment group includes chatbot. We excluded studies where both control and treatment had chatbot features. For example, the study by Liu et al [24] was excluded because all the control groups used chatbots.
4. Studies must have sufficient data provided to calculate effect sizes. This means that studies must either directly provide effect sizes in Cohen *d* or Hedges *g* or they must provide pre- and postintervention mean and SD for both treatment and control groups. The study by Maples et al [31] was excluded because of insufficient data.

### Screening

In conducting the screening process, we used Covidence software owing to its robust full-text review features and the provision of complimentary licenses available via our affiliated institutions [32]. Deduplication was handled both manually through Zotero (Corporation for Digital Scholarship) and through Covidence software. The screening of titles and abstracts, as well as the full-text review, was executed by using a double-blinded methodology to guarantee impartial evaluations. Four authors participated in the screening stage (YS, FL, CT, QZ). To resolve any conflicts, we held weekly meetings and reached a 100% consensus.

### Data Extraction and Narrative Synthesis Approach

Before data extraction, a Microsoft Excel coding framework was developed a priori. Apart from straightforward variables, intervention *duration* was extracted as the total number of weeks the intervention was delivered. If *duration* was reported as days or months, we transformed the variable into weeks using 1 week=7 days=0.25 months. To represent sex, we extracted female percentage among participants and coded *Fifty percent female* as 1 if at least 50% of the sample identified as female and 0 otherwise. The *age* variable was split into early adulthood (18–30 y old), middle adulthood (30–50 y old), and late adulthood (more than 50 y old) based on mean age. Following the framework established by Beyebach et al [33], we extracted and categorized countries as WEIRD or non-WEIRD. Studies were coded as WEIRD if they were conducted in countries that fit this classification, while those that did not meet these criteria were labeled as non-WEIRD. Studies were coded as *customized* if the chatbot allows users to customize the user interface, such as changing the app’s background color, etc. Conversely, studies were coded as *non-customized* if the chatbot did not mention *customization* in the study. Studies were coded as *clinical* if participants were recruited from healthcare or clinical service settings (eg, hospitals, outpatient clinics, or counseling centers), regardless of whether their condition was primarily mental or physical. These participants typically had documented health concerns or were receiving clinical care at baseline. By contrast, studies that recruited participants from schools, universities, or the general community without requiring a diagnosed condition were coded as *non-clinical. Outcome* measures were coded according to the primary mental health construct assessed, including depression, anxiety, and stress.

Given the heterogeneity of study designs, interventions, and outcome measures across the included studies, a narrative synthesis was conducted to summarize and compare study characteristics systematically. Following the narrative synthesis guidance from Popay et al [34], we structured the synthesis around four analytical dimensions: (1) technical features of the chatbot systems (eg, AI architecture, delivery platform, modality, customization, and embodiment), (2) treatment features (eg, theoretical frameworks, intervention duration, target outcomes, and presence of human guidance), (3) research design characteristics (eg, study type, publication year, and methods), and (4) sample characteristics (eg, country, participant demographics, population type, and recruitment setting). Each study’s information was extracted by two out of three authors independently (YS, FL, CT). Conflicts were discussed and resolved through weekly team discussions with the first author (QZ) until full agreement was reached.

### Analytic Plan

We used a random-effects model using the *metafor* package [35] in R statistical software (version 4.5.1, R Foundation for Statistical Computing). For weighted mean effect sizes, we assigned weights to each study based on inverse variance [36]. Several studies contributed multiple outcomes (eg, depression, anxiety, stress), which are statistically dependent because they are measured using the same participants. Our primary analysis used a multilevel random-effects meta-analysis with random intercepts at the study level and the outcome-within-study level [37]. The model was fit with restricted maximum likelihood (REML). To account for small-sample uncertainty in multilevel models, we used t-based inference with Satterthwaite-adjusted degrees of freedom by setting tdist=TRUE in rma.mv(). This approach provides an effect equivalent to the Hartung–Knapp–Sidik–Jonkman (HKSJ) adjustment and is particularly suitable for multivariate meta-regressions. We constructed a block-diagonal sampling variance-covariance matrix V with an assumed within-study correlation *r*=0.80. As a robustness check, we computed cluster-robust (CR2) standard errors with Satterthwaite degrees of freedom, clustering on study.

In addition to multivariate meta-analysis, as a sensitivity analysis, we fitted a univariate model using the HKSJ adjustment method. Since only 14 studies were included in the meta-analysis, we adopted HKSJ as it is recommended for meta-analysis with few studies [38]. In addition, the HKSJ method was found to outperform the standard DerSimonian-Laird method [39,40]. We fitted a random-effects model using the Sidik–Jonkman (SJ) estimator for the between-study variance (τ²), which is robust to outliers and performs well when heterogeneity is substantial. We aggregated the dataset to a single effect size per study–outcome pair. Specifically, for each study-outcome pair, we computed the mean of the reported effect sizes and the mean of their reported sampling variances. For inference on the pooled effect, we applied the HKSJ adjustment.

We assessed residual dispersion with Cochran Q and its *P* value from the fitted model. For completeness, we computed I² as the proportion of total variability attributable to heterogeneity; however, following Borenstein et al [37], we emphasize that I² is not an absolute measure of heterogeneity and does not indicate the magnitude of variation in true effects across settings. We therefore also reported τ². Given that treatment effects over different settings may vary, we also reported 95% prediction intervals (PIs) for the true effect [41].

In line with open science principles, the complete dataset and R code are publicly available [42]. We registered the protocol retrospectively during the revision process (September 18, 2025), with Open Science Framework (10.17605/OSF.IO/9DAJ7) [43]. We followed the PRISMA (Preferred Reporting Items for Systematic Reviews and Meta-Analyses) checklist. As for missing data, we either inferred from other relevant information or we reported them as NA in tables. When studies only provided post-test means and SDs, we assumed baseline equivalence and calculated Hedges *g*. When key statistics for calculating effect sizes were missing, we had to drop the study.

To evaluate publication bias, selection modeling was used. This approach used a weight function model created by Vevea and Woods [44], implemented through the *weightr* package. To assess the risk of bias for all 26 studies included in the systematic review, we adopted two Cochrane tools. For the nonrandomized studies included in the systematic reviews, we adopted the Cochrane ROBINS-I (Risk Of Bias In Non-randomised Studies – of Interventions) tool [45]. For the RCTs included in both the systematic reviews and the meta-analysis, we used the Cochrane RoB2 (revised Cochrane risk-of-bias tool for randomized trials) [46]. Two authors coded (CT, FL) independently, and a third author (QZ) resolved discrepancies.

### Moderators

Considering the need for balanced moderator categories, theoretical and practical importance, small sample size (n=14), and the need for a degree of freedom to be larger than four to ensure enough statistical power [47], we tested three moderators only. Same as the intervention effects, for the primary analysis, we applied a multilevel random-effects meta-analysis. In addition, we conducted one-moderator random-effects meta-regressions for each candidate moderator separately to avoid over-parameterization given the small number of trials. For each model, we used the SJ estimator for between-study variance and HKSJ inference for the pooled effects and moderator coefficients.

Studies were coded as *human assistance* if they included a preparatory session where a human introduced the chatbot or if human guidance was provided during chatbot use. One study, despite not explicitly mentioning human assistance, was also coded as *human assistance* based on an image showing a human assisting an older participant with ChatGPT [26]. Studies without any form of human involvement were coded as *self-guided*. Studies were coded based on social function as either *task-oriented* or *social-oriented. Task-oriented* studies were those where the chatbot’s primary function was to assist with specific tasks, such as providing information, completing exercises, or helping with specific skills (eg, learning or mental health interventions). *Social-oriented* studies were those where the chatbot’s primary function was to provide social interaction, emotional support, or companionship, without a specific focus on task completion or learning. Control group type was coded as *active* if participants received an alternative intervention, such as bibliotherapy, psychoeducation, routine care, or continued school-based support. Studies were coded as *passive* if participants received no intervention, such as waitlist control groups.

## Results

### Screening Procedures

**Figure 1.**
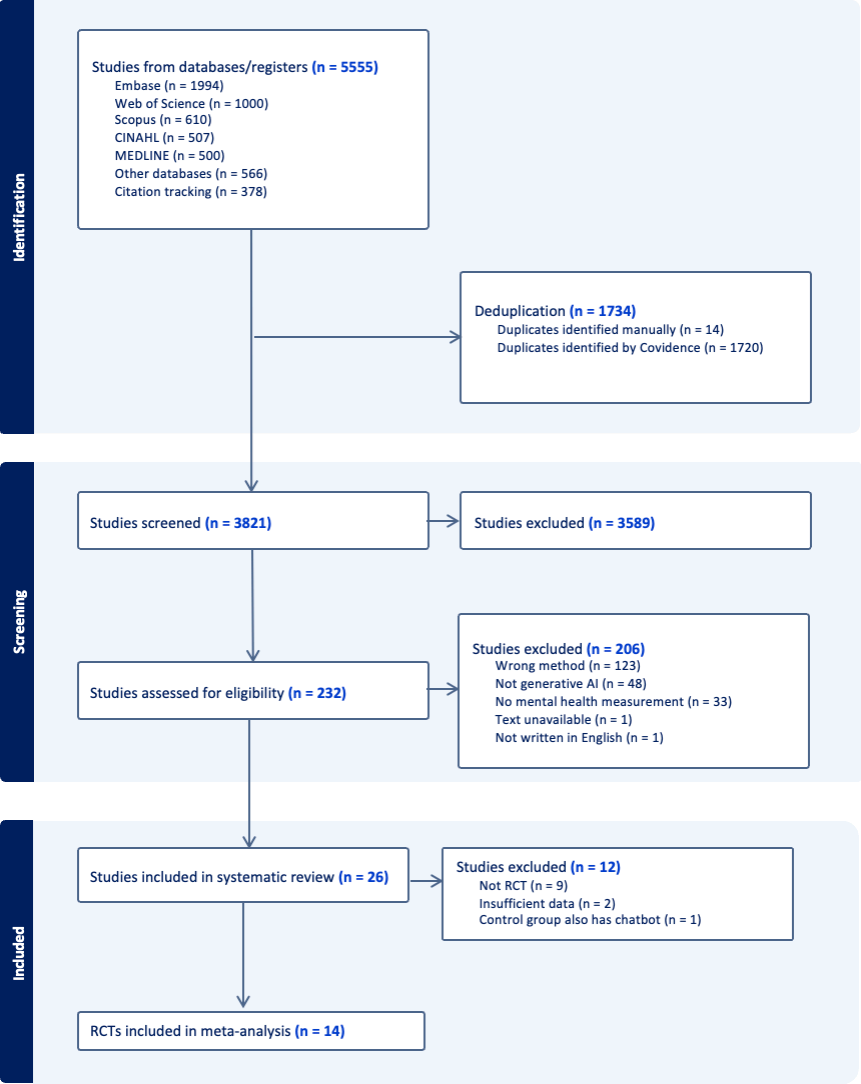
PRISMA (Preferred Reporting Items for Systematic reviews and Meta-Analyses) diagram. RCT: randomized controlled trials.

During the title and abstract screening phase and the full-text review phase, Cohen κ values were 0.5 and 0.6, indicating fair and substantial agreement, respectively. From 5555 records across databases, 26 studies met inclusion criteria for narrative synthesis. Of these, 14 RCTs (19 treatment-control comparisons, N=6314) provided sufficient data for meta-analysis (see Figure 1 for data selection process). Among the 26 studies, 1 study measured only positive well-being instead of mental health issues [29], 1 study had chatbot designs in the control group [24], 3 studies did not report sufficient data for calculating pooled effect size, and 8 studies were not randomized trials, leaving a total of 14 RCTs eligible for meta-analysis to estimate the effectiveness of GenAI chatbot on mental health issues.

### Narrative Synthesis

Table 1 presents selected major characteristics of the 26 studies included in the systematic review. Below, we provide a descriptive analysis of the interventions’ technical features, treatment features, research methods, and sample characteristics.

**Table 1.** Characteristics of the 26 studies included in the systematic review (Only 14 were included in the meta-analysis).

Study	Included in meta-analysis?	GenAI^a^ chatbot name	Response generation approach	AI^b^ technique	Customized	Interaction mode	Human assistance	Implement/integrate	Research design	Targeted outcomes	Age, mean^c^	Sample size	Female	Clinical/nonclinical Populations	Duration	Number of sessions/modules	Country
Al Mazroui & Alzyoudi, 2024 [48]	No, not RCT^d^	ChatGPT	Generative	LLM^e^	1	Text	0	No	Mixed methods	Loneliness	65.3; 60‐73	20	7	Nonclinical	2 weeks	3	United States
Liu Auren et al, 2024 [25]	No, not RCT	Replika	Generative	LSTM^f^	1	Text	0	No	Survey	Loneliness	18‐73	404	241	Nonclinical	<15 minutes, 15‐30 minutes, 10 minutes-1hour, 1-2 hours, 2-4 hours, 4 hours+ranging from daily to once a month or less	NA^g^	United States
Carl et al, 2024 [49]	No, not RCT	OpenAI GPT-4	Generative	LLM, NLP^h^, GPT	1	Text and voice	0	Medical Q&A integrated into urological counseling sessions	Pre-post intervention design	Anxiety	60.58; 18‐96	292	212	Urological patients	NA	3	Germany
Chen et al, 2025 [50]	Yes	No name	Hybrid	NLP, LLM	0	Text	0	Implemented AI chatbot to compare its effectiveness with a nurse hotline in reducing anxiety and depression among school parents in Hong Kong	RCT	Depression, anxiety	18‐60	124	NA	Nonclinical	2 weeks	2	China (Hong Kong)
Wang et al, 2024 [51]	Yes	Typebot+ EAP Talk mode 1	Generative	LLM	1	Text, voice, and video	1	An online general English-speaking course at a language institution in southeastern China	RCT	Anxiety	21; 19‐23	99	NA	Nonclinical	6 weeks	12	China
Çakmak, 2022 [52]	No, not RCT	Replika	Generative	neural network machine learning model and scripted dialogue content	1	Text and voice	0	Conducted Oral Communication Skill II course for freshmen, 2 hours per week, as a follow-up to Oral Communication Skills I.	Mixed methods	Anxiety	18‐23	90	57	Nonclinical	12 weeks	NA	Turkey
Gan et al, 2025 [53]	Yes	ChatGPT 4.0	Generative	GPT	1	Text	0	Used ChatGPT 4.0 to provide standardized responses during knee arthroplasty consent process, with physicians interpreting and contextualizing information.	RCT	Anxiety, depression, pain	72.71; 60‐80	55	43	Patients with knee osteoarthritis	2 weeks	2	China
He et al, 2022 [54]	Yes	XiaoE	Generative	NLP, deep learning	0	Text, voice, and images	0	No	Single-Blind, Three-Arm RCT	Depressive symptoms	18.78; 17‐34	148	55	Young adults with depressive symptoms	1 week	25.54	China
Heinz et al, 2024 [55]	Yes	Therabot	Generative	LLM	1	Text	0	No	RCT	Depression, anxiety, and high-risk eating disorders	33.86	210	125	Patients with depression, anxiety, or high risk of eating disorder	8 weeks	NA	United States
Habicht et al, 2024 [56]	No, not RCT	Limbic care	Generative	LLM	1	Text	0	Implemented Limbic Care AI tool in NHS Talking Therapies for Anxiety and Depression, supporting CBT exercises between sessions.	Observational study	Anxiety and depression	40.4; 18 and above	244	169	Patients with depression or anxiety receiving CBT	3 months	7	United Kingdom
Kimani et al, 2019 [57]	No, insufficient data	Angela	Hybrid	NLG^i^	1	Text, voice, and videos	1	No	Mixed methods	Confidence and anxiety	23; 18‐30	28	22	Nonclinical	NA	2	United States
Liu IV et al, 2024 [24]	No, all the control groups used chatbots	GPT-3.5 Turbo and Baidu UNIT Platform Chatbot	Generative & Retrieval	NLP, GPT-3.5	1	Text	1	No	RCT	Anxiety, the satisfaction with life, positive and negative affect, psychological well-being	18‐55	Total number: 154, sub study 1: 207, sub study 2: 70, sub study 3: 50	NA;NA;29	Nonclinical	Sub study 1: 6 days; sub study 2: 6 days; sub study 3: 2 weeks	NA	China
Liu Ivan et al, 2022 [58]	Yes	Philobot	Hybrid	NLP, sentiment analysis, BERT^j^, deep learning, rule-based retrieval, and structured decision trees	1	Text and voice	0	No	RCT Pilot Study	Resilience, happiness, positive & negative affect, depression, anxiety, mental disorder, loneliness	21.8	79	NA	Nonclinical	4 days	4	China
Drouin et al, 2022 [59]	Yes	Replika	Generative	LSTM	1	Text	0	No	3 groups experiment study	Positive & negative affect, positive & negative emotion	19.82; 18‐38	417	297	Nonclinical	20 minutes	NA	United States
Ali et al, 2024 [60]	Yes	ChatGPT, Gemini, and Perplexity	Generative	LLM	1	Text	0	No	RCT	Anxiety	18 and above	92	48	Nonclinical	4 weeks	4	Pakistan
Maples et al, 2024 [31]	No, no data	Replika	Generative	LLM, NLP, GPT	1	Text, voice, images, and videos	0	No	Cross-sectional survey study	Anxiety, social support, self-awareness, self-harm, and suicide prevention	18 and above	1006	NA	Nonclinical	NA	NA	75% were US-based, 25% were international
McFadyen et al, 2024 [61]	Yes	Limbic Care	Generative	LLM	1	Text and images	0	No	RCT	Anxiety, depression	36.84	540	NA	Patients with anxiety/depression	6 weeks	NA	UK-developed and US participants
Hu et al, 2024 [62]	No, not RCT	MyAI	Generative	LLM, GPT	1	Text	0	No	Within-subject experimental design	Negative affect, stress, social support, loneliness	21; 18‐29	150	118	Nonclinical	3 weeks	2	Singapore
Romanovskyi et al, 2021 [63]	Yes	Elomia	Generative	LLM: RoBERTa(NER)	0	Text	0	No	RCT	Depression, anxiety	21; 19‐23	82	39	Nonclinical	4 weeks	NA	Ukraine
Sabour et al, 2023 [64]	Yes	Emohaa (ES-Bot+CBT^k^-Bot)	Hybrid	NLP, LLM	0	Text	1	No	RCT	Depression, anxiety, positive & negative affect, insomnia	30.9	247	190	Nonclinical	3 weeks	7	China
Zheng, 2024 [30]	No, not RCT	Reading Bot	pretrained LLM	LLM	1	Text	1	Used AI chatbot to clarify reading confusion in junior secondary EFL class.	Quasi-experimental pre-test/post-test.	Anxiety	12.845; 11‐14	84	46	Nonclinical	3 hours 45 minutes	5	China
Vowels et al, 2024 [29]	No, positive outcomes	Amanda	Generative	LLM, GPT	1	Text	0	No	RCT	Well-being, distress, hopefulness, confidence, satisfaction, self-demand/partner-withdraw, partner-demand/self-withdraw	36.6	258	169	Nonclinical	NA	1	United Kingdom
Wang & Farb, 2024 [65]	Yes	No name	Generative	LLM	1	Text	0	No	RCT	Wellness, mindfulness	18.86; 17‐40	114	83	Nonclinical	1 week	7	Canada
Wang & Li, 2024 [26]	No, not RCT	ChatGPT-3.0	Generative	GPT	1	Text and voice	1	No	Controlled design	Loneliness, depression, and life satisfaction	80.4	12	1	Nonclinical	8 weeks	8	China
Yahagi et al, 2024 [66]	Yes	ChatGPT-3.5	Generative	GPT	0	Text	0	Patients interacted with ChatGPT for preoperative information about Anesthesia	RCT	Anxiety	57.5	85	42	Nonclinical	4 weeks	4	Japan
Zheng et al, 2025 [67]	Yes	No name	Generative	LLM, GPT	1	Text and voice	1	Adapted Wang’s (2014) four-stage model for English speaking, integrating LLM functionality into the experimental group	RCT	Anxiety	18.66	83	57	Nonclinical	5‐7 days	1	China

a GenAI: generative artificial intelligence.

b AI: artificial intelligence.

c Range is provided, if available.

d RCT: randomized controlled trial.

e LLM: large language model.

f LSTM: long short-term memory.

g NA: not available.

h NLP: natural language processing.

i NLG: natural language generation.

j BERT: bidirectional encoder representations from transformers.

k CBT: cognitive behavioral therapy.

### Technical Features

Among the 26 included studies, 21 used purely GenAI and 5 used hybrid models combining generative and rule or retrieval-based approaches. Among the 11 studies utilizing GenAI, all systems were based on large language model (LLM) architectures, within which three studies specifically used GPT-series models. In addition, eight studies integrated LLM with other AI techniques, such as natural language processing (NLP). Two studies used long short-term memory (LSTM) models [25,59], one incorporated NLP with dynamic programming (DP) [54], one study used neural network machine learning model and scripted dialogue content [52], one incorporated NLP alongside GPT-3.5 [24], another integrated NLP, BERT, deep learning [58], and one used natural language generation (NLG) [57].

Most studies used a single AI chatbot, including ChatGPT (various versions, n=5), Replika (n=4), Limbic care (n=2), and one study each for Elomia [63], Philobot [58], MyAI [62], Virtual Coach Angela [57], Reading Bot [30], Emohaa [64], Amanda [29], XiaoE [54], and Therabot [55]. Three studies explored multiple AI chatbots, such as ChatGPT, Gemini, and Perplexity [60], or Typebot/ D-ID Agent and EAP Talk [51], or GPT-3.5 Turbo and Baidu UNIT [24] platform chatbot. Three studies did not specify a chatbot name.

Regarding delivery format, the majority (n=11) required the use of a smartphone only, 6 used only web-based platforms (including two web applications), 2 used both smartphone and web-based platforms and 3 studies did not specify the platform. As for interaction mode, all 26 studies used text-based interaction, with 11 studies incorporating voice features and six studies further including image-based interactions.

All studies used nonembodied AI chatbots, except for two that incorporated embodied virtual agents [51,57]. Furthermore, among the 26 studies, 21 implemented AI chatbots with customized features.

### Treatment Features

The duration of chatbot intervention ranged from 20 minutes [59] to 3 months [56] (mean 3.63, SD 1.74 wk). Nine studies explicitly incorporated cognitive behavioral therapy principles, while one drew from positive psychology [58] and two from mindfulness interventions [26,65]. However, the rest of the studies (n=14) did not specify the guiding theoretical model.

Interventions targeted outcomes varied, including mental health concerns (eg, depression, anxiety, insomnia, stress), social well-being (eg, loneliness, social support), school-, language-, or test-related anxiety, and anxiety related to medical procedure (eg, preoperative anxiety, hospital anxiety).

Most studies (n=18) incorporated some form of human support alongside chatbot use, such as clinician facilitation [56] and teacher supervision [51]. In contrast, eight studies featured fully autonomous AI chatbots. It seems like AI chatbots can assist in-person counseling sessions but might not replace human therapy. For example, one study found that the GenAI support system significantly improved patient attendance and treatment outcomes [68]. However, a study also found that mental health chatbot alone did not outperform the traditional bibliotherapy method in developing [58] participants’ resilience.

A few studies embedded chatbots into structured health care (n=5), including urological counseling for urological surgery [49], preoperative patient education [66], therapy support between sessions [23], knee arthroplasty patients’ consent process [53], assistance to health care professionals in a conventional school nurse hotline [53]. Some studies embedded chatbots in educational settings (n=3), such as oral communication skills courses for university freshmen [52], junior secondary English as a Foreign Language classes’ activities for clarifying reading confusion [30], and online general English-speaking courses [51].

### Research design

**Figure 2.**
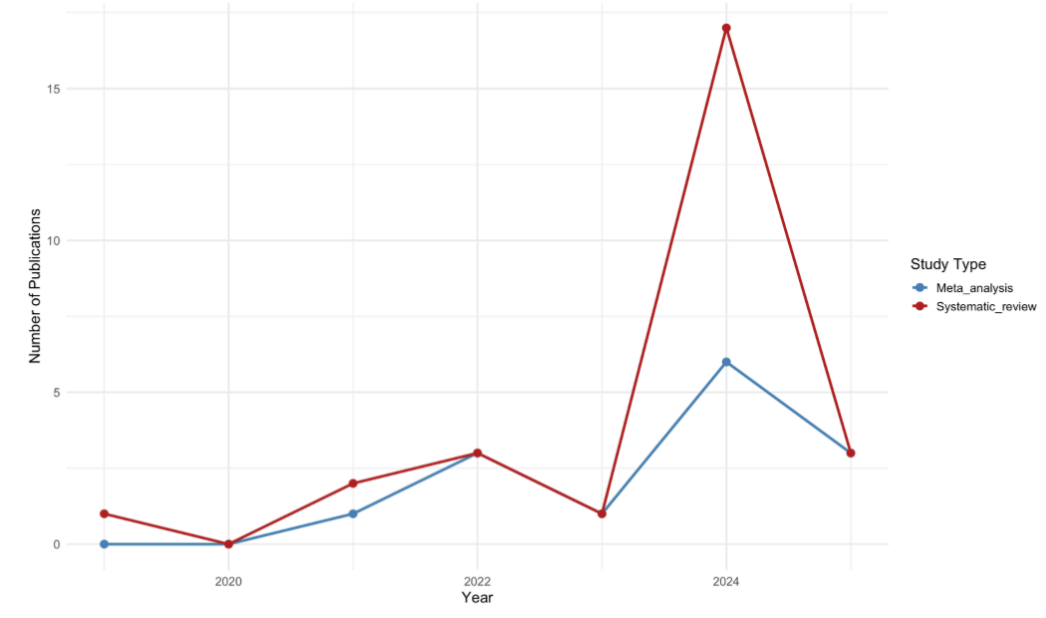
Number of studies published per year. The chart illustrates the number of systematic review and meta-analysis publications from 2019 to 2025. Data represent the total number of studies published each year, based on the inclusion criteria established for this review. Note that the literature search concluded in March 2025, which explains the drop in 2025.

Fifteen studies used RCTs, while the remainder used quasi-experimental (n=5), mixed-methods (n=3), survey (n=2) [26], and observational design (n=1) [23]. Publication years ranged from 2019 to early 2025 (March when the search concluded), with 17 studies published in 2024 alone, which resonates with AI’s exponential growth. Figure 2 presents a plot of the number of studies published per year.

### Sample Characteristics

Collectively, a total of 5469 participants from 11 countries and regions were involved. Most were single-site studies, with 10 conducted in China, 6 in the United States, 3 in the United Kingdom [26,37,61], and 1 each in Turkey [52], Germany [49], Singapore [62], Pakistan [60], Ukraine [63], Canada [65], and Japan [66]. Out of the 10 studies conducted in China, 2 used English-language AI platforms because they targeted English learning among Chinese students [30,67], and the remaining 8 studies used Chinese. Based on Beyebach et al’s [33] WEIRD versus non-WEIRD framework, 15 out of 26 studies (58%) were conducted in non-WEIRD countries.

Exactly half of the studies [55] (n=13, including one study recruited partially students and partially nonstudents) focused on student populations, while the remainder involved general adult participants (n=13). The participants’ age ranged from 11 [30] to 96 [49] years. Meanwhile, 21 out of 26 studies (81%) from the systematic review focus on early- and middle-aged adults (18–50 y old). Only one study focused on adolescents exclusively [41], and three studies focused on older adults [48,53,66]. The studies’ sample size ranges all the way from 12 [26] to 1006 [31] (mean 198.85, SD 210.68). Most studies (n=20) involved nonclinical participants, while six recruited clinical populations, including patients attending urological counseling for elective surgery [49] (n=1), people experiencing symptoms of depression or anxiety [16,23,61] (n=3), people with high-risk eating disorders and other mental health conditions (n=1) [55], or knee osteoarthritis patients (n=1) [53].

### Intervention Effects and Sensitivity Analyses

**Figure 3.**
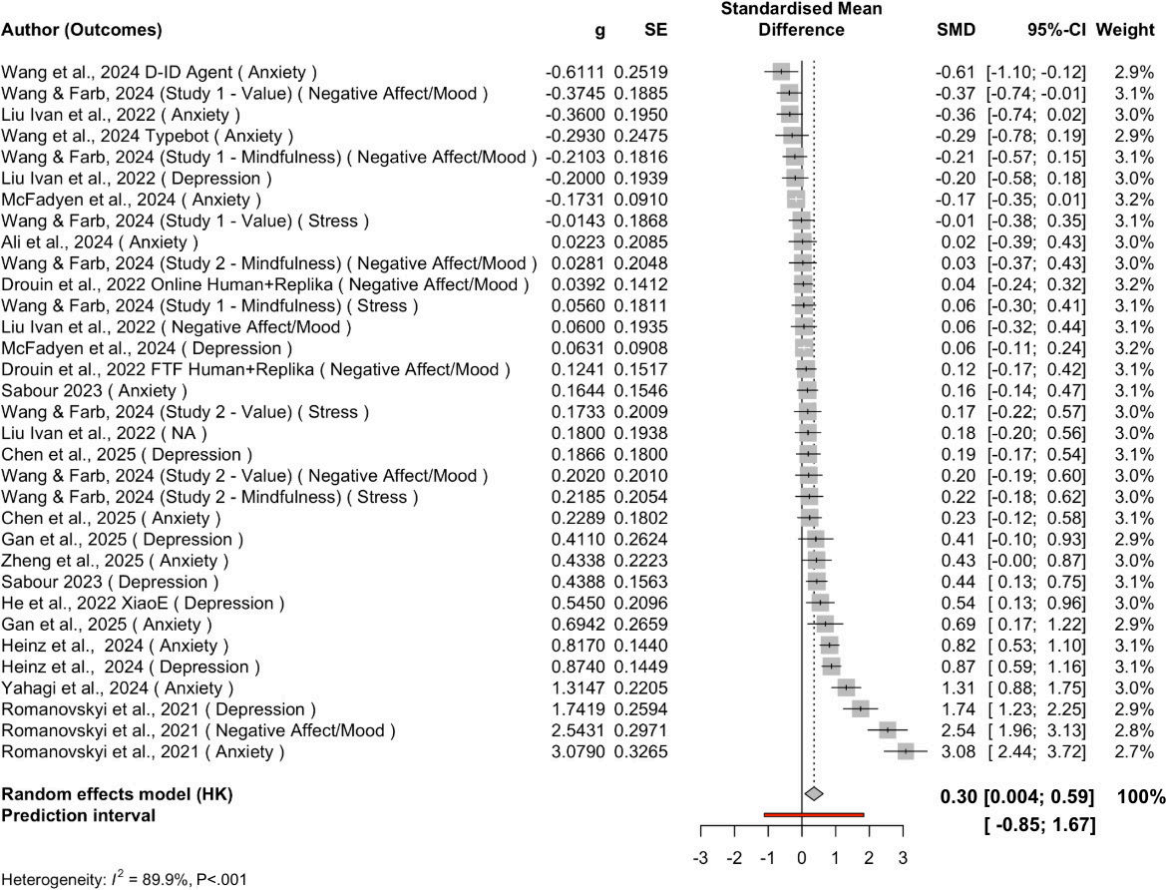
Forest plot for all outcomes. Studies were organized from smaller SMDs to larger SMDs [26,50,51,53-55,58-61,63-67]. g: Hedges *g*; PI: predictive intervals; SE: standard error; SMD: standardized mean difference.

Table 2 presents the descriptive statistics of the 14 RCT studies with 19 treatment-control pairs (n=6314) included in the meta-analysis. Figure 3 presents the forest plot of these 14 studies’ effect sizes and outcomes.

**Table 2.** Descriptive statistics of the 13 studies included for meta-analysis (14 studies include 19 treatment-control pairs). We have 14 articles but 19 treatments. Therefore, for the sake of analysis, 18 was used for meta-analysis.

Category	Level	Overall (%)
Study level		
Total treatments (n=19)		
WEIRD^a^		
No		11 (57.9)
Yes		8 (42.1)
Clinical populations		
No		14 (73.7)
Yes		5 (26.3)
Age^b^		
Early adulthood		12 (63.2)
Late adulthood		2 (10.5)
Middle adulthood		4 (21.1)
NA^c^		1 (5.3)
Sex		
Less than 50% female		9 (47.4)
More than 50% female		10 (52.6)
Customized		
No		6 (31.6)
Yes		13 (68.4)
Human assistance		
Purely self-guided program		15 (78.9)
With human assistance		4 (21.1)
Modality		
Hybrid		7 (36.8)
Text-based		12 (63.2)
Social function		
Social-oriented		7 (36.8)
Task-oriented		12 (63.2)
Outcome level		
Total effect sizes (n=44)		
Outcomes		
Anxiety		19 (43.2)
Depression		12 (27.3)
Loneliness		1 (2.3)
Negative mood or Affect		8 (18.2)
Stress		4 (9.1)
Clustered		
0		38 (86.4)
NA		6 (13.6)
Control group		
Active		28 (63.6)
Passive		16 (36.4)
Follow-up		
Without follow-up assessments		37 (84.1)
With follow-up assessments		7 (15.9)

a WEIRD is an acronym for Western, Educated, Industrialized, Rich, and Democratic following Beyebach et al’s [33] framework.

b Age was split into three categories: early adulthood (18–30 y old), middle adulthood (30–50 y old), and late adulthood (more than 50 y old) based on mean age.

c Not available.

Table 3 presents the multilevel meta-analysis including 44 effects across 14 study clusters. The overall pooled effect was 0.30 (SE 0.14), *P*=.047, with a 95% CI (0.004, 0.59) and 95% PI (−0.85 to 1.67) under the REML estimator. This corresponds to a small-to-moderate positive effect of chatbot-based mental health interventions compared to control groups. The 95% PI indicated wide real-world variability in effects, suggesting that true effects across similar contexts could range from negligible to moderately large in magnitude. Between-study heterogeneity was substantial (σ²=0.332, *τ*=0.576), with additional within-study residual variation at the outcome level (σ²=0.039, *τ*=0.198). The residual heterogeneity test confirmed significant dispersion (Q [35]=230.41, *P*<.001).

**Table 3.** Multivariate random-effects meta-regression model results for models with and without moderators.

Coefficient	SMD^a^	SE^b^	*t* test	*df* ^c^	*P* value	95% PI^d^
Null model for all outcomes						
Intercept	0.30	0.14	2.13	17.9	.047^e^	−0.85, 1.67
Null model for depression						
Intercept	0.49	0.20	2.5	6.97	.04^e^	−0.51, 1.54
Null model for anxiety						
Intercept	0.43	0.28	1.56	11	.15	−1.08, 2.05
Null model for negative affect and mood						
Intercept	0.28	0.31	0.92	7	.39	−1.95, 2.52
Null model for stress						
Intercept	0.10	0.05	1.92	2.96	.15	−0.31, 0.51
Single predictor model with one moderator						
Social function: task-oriented (as compared to social-oriented)	−0.78	0.28	−2.76	12.45	.02^e^	—^f^
Single predictor model with one moderator						
Human assistance (as compared to self-guided)	−0.39	0.29	−1.34	4.63	.24	—
Single predictor model with one moderator						
Passive control group (as compared to active control group)	0.202	0.25	0.82	4.78	.45	—
Full model with three moderators						
Intercept	0.77	0.34	2.28	5.74	.06	—
Passive control group (as compared to active control group)	0.07	0.24	0.31	4.90	.77	—
Human assistance (as compared to self-guided)	−0.03	0.25	−0.12	5.812	.91	—
Social function: task-oriented (as compared to social-oriented)	−0.76	0.32	−2.38	11.26	.04^e^	—

a SMD: standardized mean difference.

b SE: standard error.

c df: degrees of freedom.

d PI: predictive intervals.

e *P*<.05.

f Not available.

As for sensitivity analysis, we performed two changes: (1) we changed the *r* specification between 0.2 to 0.8 and the result is robust, and (2) we applied univariate HKSJ-SJ random-effects meta-analysis, and the conclusion remains the same. Details of the univariate HKSJ-SJ meta-regression model results for models with and without moderators can be found in MultimediaAppendices 5  6.

**Figure 4.**
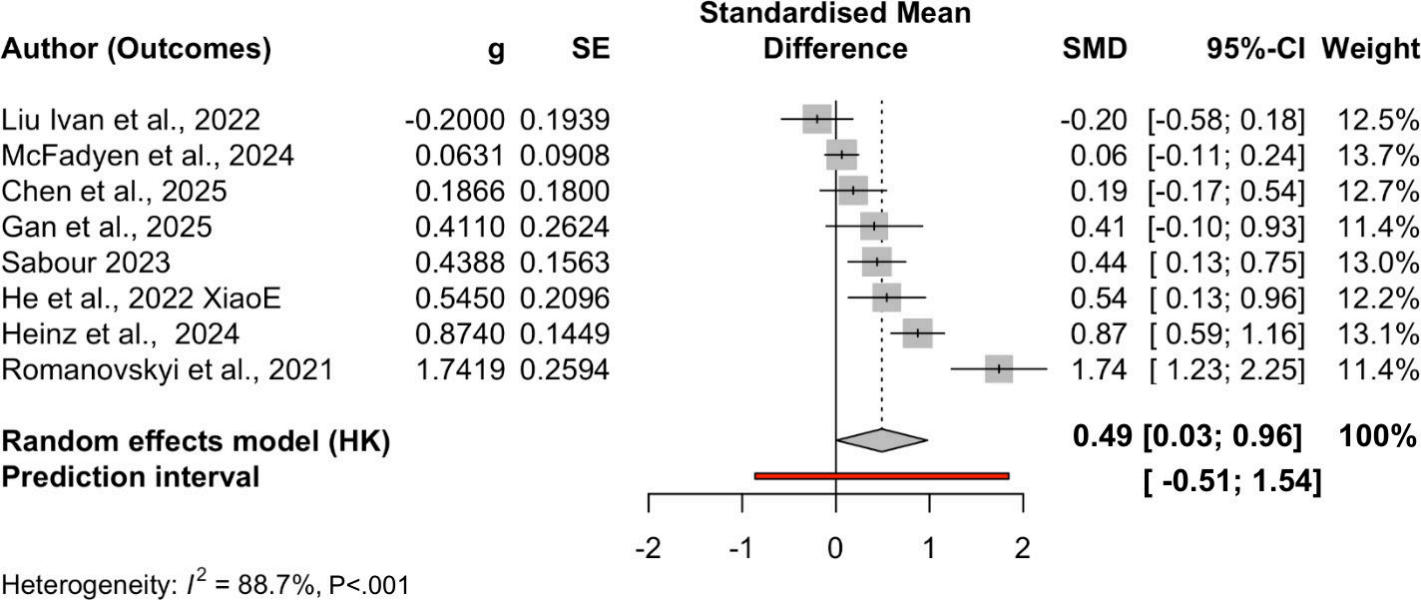
Forest plot for depression outcomes. Note studies were organized from smaller SMDs to larger SMDs [50,53-55,58,61,63,64]. g: Hedges *g*; PI: predictive intervals; SE: standard error; SMD: standardized mean difference.

Apart from weighted average effects across all included studies, we also conducted subgroup analyses by outcome (Table 3). The pooled effect for depression (k=12) was 0.49 (SE 0.20; *P*=.04, 95% CI 0.03, 0.96, 95% PI −0.51, 1.54), with high heterogeneity (Q[11]=68.23; *P*<.001; τ²=0.225, I² ≈ 90%). The pooled effect for anxiety (k=19) was 0.43 (SE 0.28; *P*=0.15, 95% CI −0.18, 1.03, 95% PI (−1.08, 2.051), also with substantial heterogeneity (Q[18]=142.63, *P*<.001; τ²=0.857). The pooled effect for negative affect or mood (k=8) was 0.28 (SE=0.31; *P*=.39, 95% CI −0.45, 1.02, 95% PI −1.95, 2.52), with very high heterogeneity (Q[7]=77.00, *P*<.001; τ²=0.664). The pooled effect for stress (k=4) was 0.10 (SE=0.05: *P*=.15, 95% CI –0.21, 0.41, 95% PI −0.31, 0.51, with negligible heterogeneity (Q[3]=0.90, *P*=.83; τ²=0). There is only one effect size on loneliness; therefore, we skipped subgroup analysis on this outcome. Figures4^7^ present forest plots for four subgroup outcomes.

**Figure 5.**
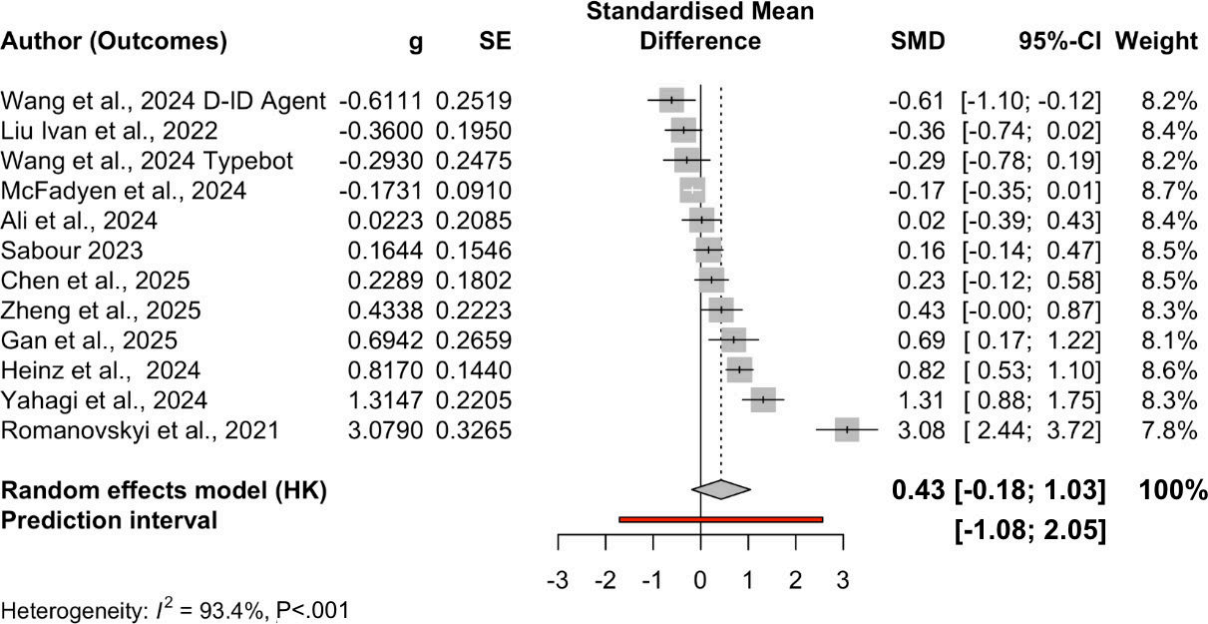
Forest plot for anxiety outcomes. Note studies were organized from smaller SMDs to larger SMDs [26,50,51,53,55,58,60,61,63,64,66,67]. g: Hedges *g*; PI: predictive intervals; SE: standard error; SMD: standardized mean difference.

**Figure 6.**
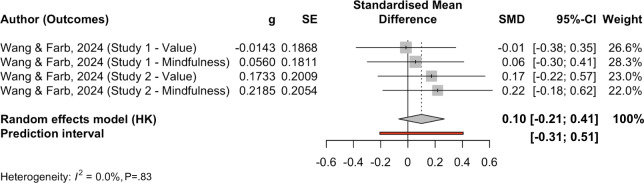
Forest plot for stress outcomes. Note studies were organized from smaller SMDs to larger SMDs [65]. g: Hedges *g*; PI: predictive intervals; SE: standard error; SMD: standardized mean difference.

**Figure 7.**
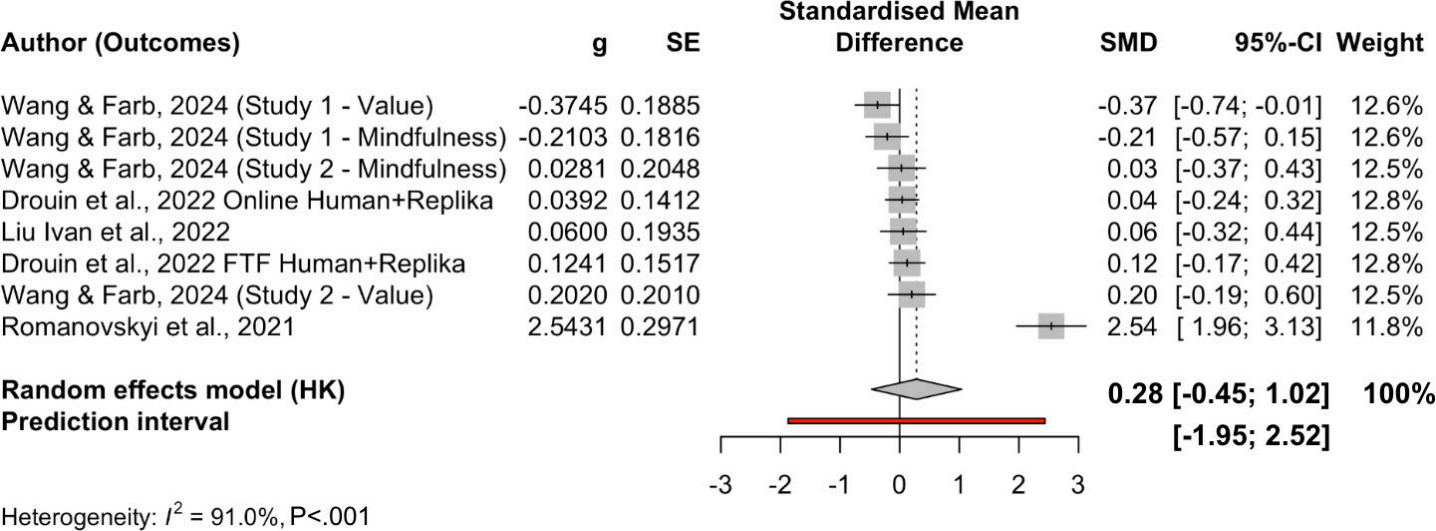
Forest plot for negative affect and mood outcomes. Studies were organized from smaller SMDs to larger SMDs [58,59,63,65]. g: Hedges *g*; PI: predictive intervals; SE: standard error; SMD: standardized mean difference.

**Figure 8.**
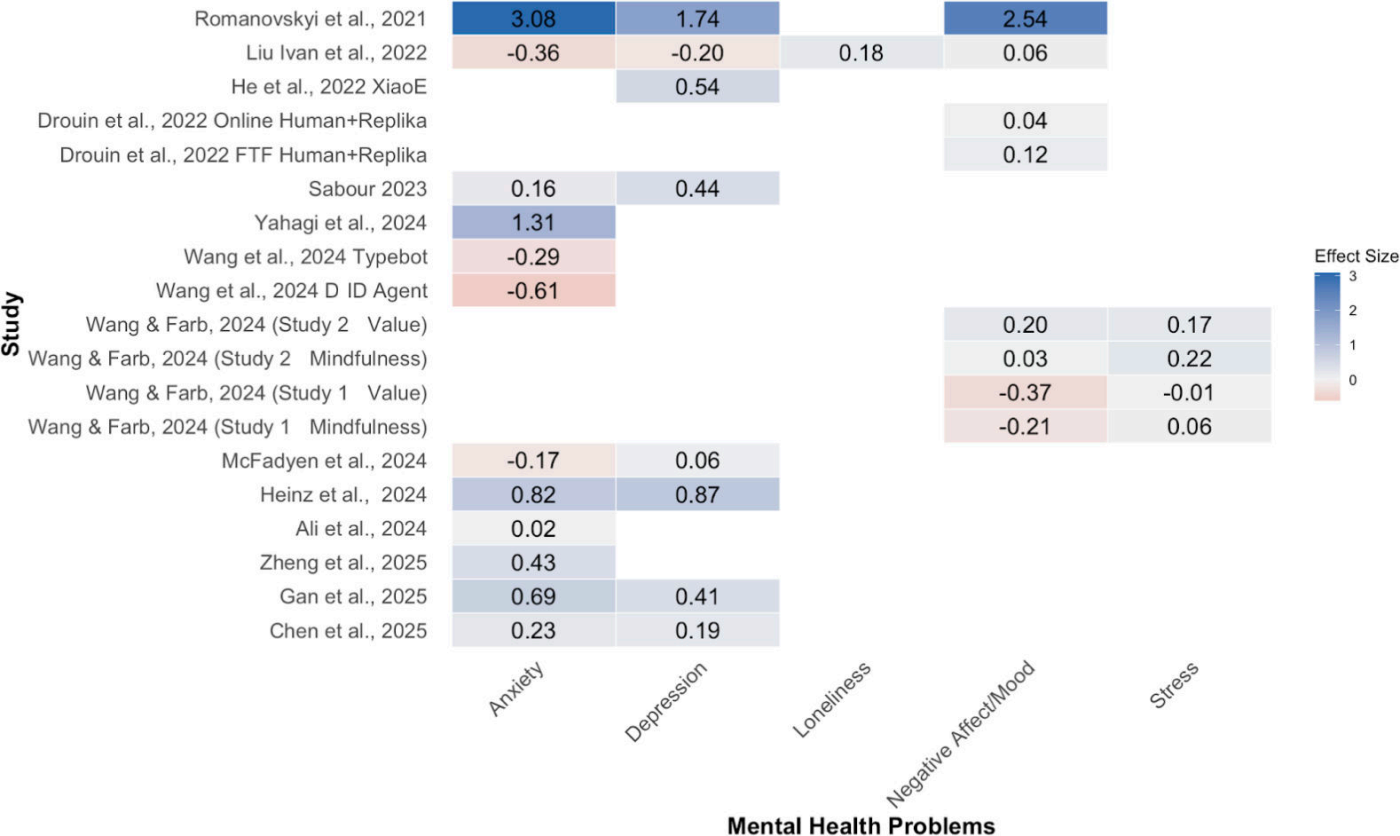
Heatmap of each study’s outcome and effect size. Note the studies were organized chronologically. For effect sizes, darker color means higher absolute values, with blue indicating positive effect sizes and red indicating negative effect sizes [50,51,53-55,58-61,63-67].

Among these, only the depression subgroup showed a statistically significant positive effect. The wide PIs, especially for anxiety and negative affect or mood, indicate that true effects in new, similar settings may range from negligible or unfavorable to moderately beneficial. Readers must be cautious since some subgroups have a small number of studies. Figure 8 complements the subgroup findings with a heatmap of each study’s outcome and effect size.

### Moderator Analysis

The combined moderator model (Active/Passive+Human Assistance+Social Function) was significant overall (F(3, 36)=3.11, *P*=.04). Within this model, social function is a strong predictor (SMD=−0.76, *P*=.04), whereas human assistance (SMD=−0.03, *P*=.91) and active versus passive control group (SMD=0.07, *P*=.77) did not influence the effect. Specifically, task-oriented chatbots showed smaller effects (SMD=0.007, SE=0.06, *P*=.91) compared to socially oriented chatbots (SMD=0.77, SE=0.34, *P*=.06).

**Figure 9.**
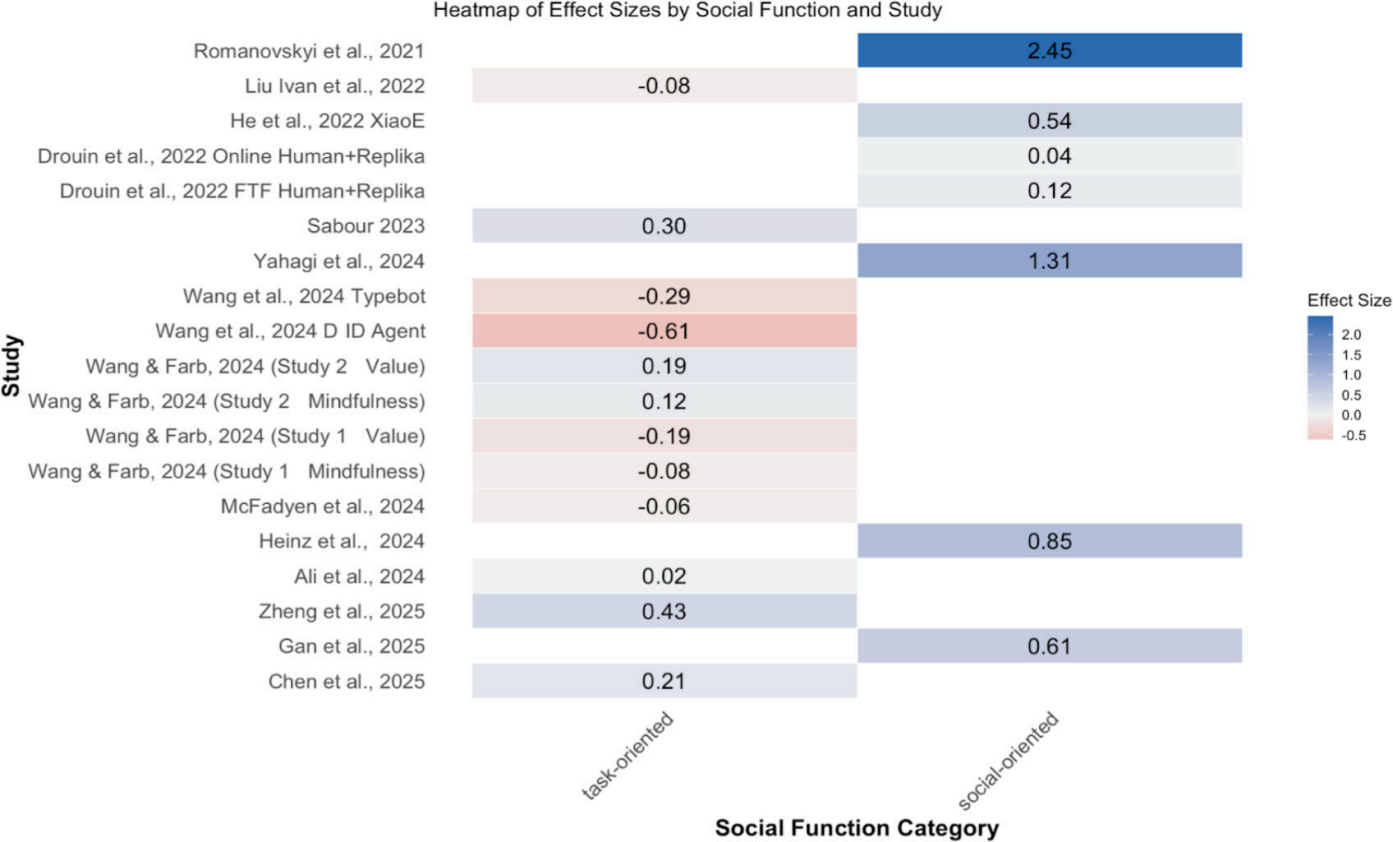
Heatmap of each study’s effect size by social function and study. The studies were organized chronologically. For effect sizes, darker color means higher absolute values, with blue indicating positive effect sizes and red indicating negative effect sizes [50,51,53-55,58-61,63-67].

The sensitivity analyses using one-moderator univariate random-effects models with SJ τ² and HKSJ confirmed the results from the multivariate model that task-oriented chatbots are less effective as compared to social-oriented chatbots. Figure 9 presents a heatmap of each study’s effect size by social function and study, which clearly shows that social-oriented chatbots are consistently found with more positive effect sizes across different studies.

### Selection Bias

**Figure 10.**
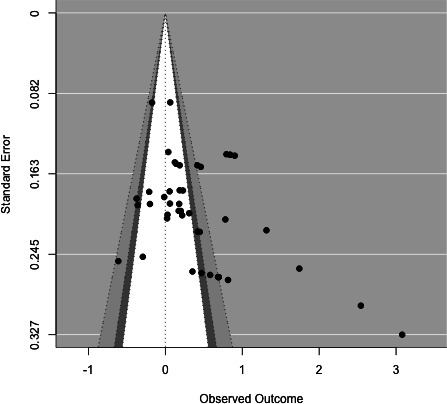
Funnel plot.

Figure 10 presents a funnel plot. Visual assessment reveals that there is asymmetry. We then applied Vevea and Woods’ [44] weight-function model to assess potential publication bias. The unadjusted model (k=44) estimated a pooled effect size of g=0.41 (SE=0.10, z=4.24; *P*<.001, 95% CI 0.22, 0.60). After adjustment for potential selection bias, the two-step model (*P* value cutpoints=.025, .50, 1) yielded a smaller and nonsignificant change in the pooled effect (g=0.54, SE=0.26, z=2.10; *P*=.04, 95% CI 0.04, 1.04). The estimated weights indicated that studies with *P*<.025* were about 3.54 times more likely to be included than those with larger *P* values, suggesting moderate publication bias favoring statistically significant findings. The likelihood ratio test comparing adjusted and unadjusted models was significant (*χ*²_2_=11.25, *P*=.004), confirming evidence of selection bias.

### Risk of Bias Analysis

Table 4 presents the risk of bias assessment from the Cochrane tool, ROBINS-I for nonrandomized studies included in systematic review but excluded from meta-analysis. This tool assesses the risk of bias in nonrandomized studies across seven domains: (1) confounding, or whether external factors influenced outcomes; (2) selection of participants, assessing if inclusion or exclusion introduced bias; (3) classification of interventions, evaluating accurate group assignment; (4) deviations from intended interventions, considering adherence and co-interventions; (5) missing data, addressing loss and its impact; (6) measurement of outcomes, assessing objectivity and consistency; and (7) selection of the reported result, evaluating selective outcome reporting. Overall, the risk of bias ranges from moderate to serious, and 66.67% (8/12) studies were ranked as serious risk.

**Table 4.** Risk of bias using ROBINS-I (Risk Of Bias In Non-randomised Studies – of Interventions) for nonrandomized studies.

Study	Domain 1 confounding	Domain 2 classification of interventions	Domain 3 selection of participants	Domain 4 deviations from intended interventions	Domain 5 missing data	Domain 6 measurement of outcomes	Domain 7 selection of the reported result	Overall risk of bias
Al Mazroui & Alzyoudi, 2024 [48]	S^a^	L^b^	S	M^c^	M	M	M	S
Çakmak, 2022 [52]	S	M	M	M	M	M	M	S
Carl et al, 2024 [49]	L	L	M	M	L	M	L	M
Habicht et al, 2024 [56]	S	M	M	M	L	M	L	S
Hu et al, 2024 [62]	L	L	L	M	L	M	L	M
Kimani et al, 2019 [57]	L	L	L	L	L	M	L	M
Liu Auren et al, 2024 [25]	L	L	L	M	L	M	L	M
Liu IV et al, 2024 [24]	L	L	M	M	M	M	M	S
Maples et al, 2024 [31]	S	L	M	L	M	M	M	S
Vowels et al, 2024 [29]	L	L	L	L	L	M	M	S
Wang and Li, 2024 [26]	S	L	M	M	S	M	L	S
Zheng, 2024 [24]	S	L	L	M	L	M	L	S

a S: serious risk.

b L: low risk.

c M: moderate risk.

Table 5 presents the Cochrane RoB2 for the RCTs included in meta-analysis. This tool assesses included studies in five domains: (1) bias arising from the randomization process, (2) bias due to deviations from intended interventions, (3) bias due to missing outcome data, (4) bias in measurement of the outcome, and (5) bias in selection of the reported result. Domain 1 was assessed at the study level, whereas the other domains were assessed at the result level. Each domain was rated as low risk of bias, some concerns, or high risk of bias, and an overall judgment for each study was derived based on these domain-level assessments. The results showed that 64.29% (9/14) of studies were rated as having some concerns and 35.71% (5/14) as high risk, with no study judged to be at low risk of bias.

**Table 5.** Risk of bias using the Cochrane RoB2 for randomized control trials (RCTs).

Study	Domain 1 Randomization	Domain 2 Deviation from intended interventions	Domain 3 Missing outcome data	Domain 4 Measurement of the outcomes	Domain 5 Selection of the reported results	Overall risk of bias
Ali et al, 2024 [60]	L^a^	L	L	SC^b^	L	SC
Chen et al, 2025 [50]	SC	H^c^	SC	SC	SC	H
Drouin et al, 2022 [59]	H	SC	L	H	L	H
Gan et al, 2025 [53]	L	SC	L	L	L	SC
He et al, 2022 [54]	L	L	L	SC	L	SC
Heinz et al, 2024 [55]	L	SC	L	SC	L	SC
Liu Ivan et al, 2022 [58]	SC	SC	H	SC	L	H
McFadyen et al, 2024 [61]	SC	SC	L	SC	L	SC
Romanovskyi et al, 2021 [63]	L	H	SC	SC	SC	H
Sabour, 2023 [64]	L	L	SC	SC	SC	SC
Wang & Farb, 2024 [65]	L	SC	L	L	L	SC
Wang et al, 2024 [51]	L	SC	SC	SC	SC	SC
Yahagi et al, 2024 [66]	L	H	SC	SC	L	H
Zheng et al, 2025 [67]	L	SC	L	SC	SC	SC

a L: low risk.

b SC: some concerns.

c H: high risk.

## Discussion

### Principal Findings

This review provides the first systematic synthesis and meta-analysis focused on GenAI chatbots for mental health outcomes, including 26 articles in the systematic review and 14 RCTs in meta-analysis. Overall, our results indicate a small-to-moderate but statistically significant average effect, suggesting that GenAI mental health chatbot interventions may be effective in reducing mental health issues. However, wide prediction intervals and substantial between-study heterogeneity indicate that these benefits are not consistent across studies or populations. This was similar to previous meta-analyses on the effectiveness of rule-based, retrieval-based, and GenAI chatbots [13,15,16,18]. Yet, it is crucial to note that effectiveness varies depending on chatbots’ design and target outcome.

### Social-Oriented Chatbots Are More Effective Than Task-Oriented Chatbots

Social function emerged as the most consistent moderator across different models. We found that social-oriented chatbots are more effective than task-oriented chatbots, although this result should be interpreted cautiously given the limited number of included studies and the high heterogeneity of effects. This pattern aligns with previous literature on social chatbots leading to better consumer satisfaction [69] and social outcomes for older adults [70]. Decades of research demonstrate that perceived social support is a protective factor against stress, depression, and anxiety [71,72]. Social chatbots can simulate supportive relationships, offering emotional validation, empathy, and companionship, even if users cognitively recognize the artificiality of the interaction [73]. This aligns with the Computers Are Social Actors (CASA) paradigm [74], which shows that humans often respond to machines using the same social heuristics they apply to human partners. In contrast, task-oriented chatbots, lacking this socio-emotional dimension, primarily provide informational rather than emotional support, limiting their impact on distress.

The effect may also be because social interactions with AI chatbots facilitate therapeutic alliance, one of the most effective factors in psychotherapy [75,76]. From psychotherapy research, the common factors model emphasizes that therapeutic alliance, empathy, and relational depth are among the strongest predictors of positive clinical outcomes [77,78]. Social chatbots, by offering empathic, personalized, and emotionally attuned exchanges, can foster trust and disclosure, which may help reduce negative mental health issues [69]. Task-oriented chatbots, by contrast, often lack the flexibility to respond empathetically, limiting their capacity to generate the relational bonds essential for emotional relief.

An important implication of this finding is that developers should consider integrating relational design principles, including empathy, warmth, and social support, into conversational systems. Designing AI interactions that communicate acceptance and genuine care may enhance users’ emotional engagement and psychological well-being, aligning chatbot interactions more closely with the therapeutic mechanisms that underpin effective human support.

### Outcome Subgroup: GenAI Chatbots Are Most Effective in Treating Depression

Among the outcome subgroups (depression, anxiety, stress, negative moods), effect sizes were positive across all groups, but only the depression subgroup demonstrated a statistically significant effect (ES=.49, *P*=.041). Depression and anxiety are the most studied outcomes in existing studies of GenAI mental health chatbots. This finding is unsurprising, given that both disorders are not only the most prevalent [79] but also highly comorbid [80]. While our results suggest GenAI chatbots’ promise in addressing depression, these technologies should be positioned as supplementary instead of replacement treatments. Depression management typically requires long-term care, and approximately half of patients relapse after an initial episode [81]. Effective treatment requires careful examination of patients’ medical history, symptom trajectory, and sustained therapeutic alliances, which cannot be fully replicated by current GenAI systems. Therefore, GenAI chatbots might act as complementary supports to enhance counselors’ efficiency and extend access to care. Indeed, in our systematic review, 69.23% of GenAI interventions incorporated some forms of human assistance instead of relying solely on fully autonomous GenAI chatbot experience. Future studies could explore how to deliver more targeted, personalized, and sustainable treatment through optimal combination of human expertise with GenAI technology.

In contrast, despite plenty of studies focusing on depression and anxiety, there was a severe lack of studies focusing on negative mood and stress. This imbalance reflects a broader gap in the literature, particularly regarding the role of GenAI chatbots in managing more severe or complex mental health conditions. The increasing severity and complexity of global mental health challenges highlight the limited application of chatbots to severe mental health conditions such as suicidality, schizophrenia, or substance use disorders. Taken together, these gaps indicate that while GenAI chatbots may serve as valuable adjuncts to care, they should not be viewed as standalone solutions for addressing the full spectrum of mental health needs. Rather, their role lies in complementing human-delivered services and expanding access to support, especially in contexts where resources are scarce.

### Most Interventions Took Place in WEIRD Countries

Most of the studies (58%, 15/26) took place in non-WEIRD countries, such as China. While comparing across continents, there is a severe lack of GenAI chatbot studies from Europe. One explanation could be the stringent and comprehensive AI regulation in European countries introduced by the European Union [82]. The self-regulatory AI market in other countries, such as China, United States, and United Kingdom, might help the local AI development in mental health areas. However, these cross-national observations are descriptive rather than inferential; our study did not test the effects of cultural or regulatory differences statistically. Large language models are frequently trained on datasets predominantly sourced from WEIRD contexts [83]. Results suggested that there are some systematic differences between WEIRD and non-WEIRD countries in terms of age and recruitment type. Consequently, when these models are deployed in non-WEIRD contexts, they may not fully grasp or appropriately respond to culturally specific nuances or local dialects.

With the global shortage of mental health resources, especially in non-WEIRD countries, it is essential to examine how cultural differences shape the adoption and effectiveness of GenAI chatbots for mental health [84]. Cultural beliefs and stigma influence willingness to seek digital support, while differences in language and communication styles affect the perceived appropriateness of chatbot responses [85,86]. Training AI systems with culturally representative data and considering local ethical and regulatory contexts may improve trust, relevance, and uptake [87]. Attention is needed regarding the generalizability of the intervention results across diverse cultural and socioeconomic contexts. Further research is needed to adapt these interventions to non-WEIRD contexts, taking into account local cultural nuances and resource availability [88].

### Lack of Studies on Adolescents, Older Adults, and Applications in Diverse Settings

Eighty-one percent of studies from the systematic review focus on early- and middle-aged adults (18–50 y old), with only one study investigating adolescents (<18 y old) and three studies focusing on the older adults (>50 y old). This might be attributed to a cautious attitude towards GenAI’s impact on youth and the lack of research focus on older adults because of the potential concern regarding their technological skills. In the increasingly aging society, GenAI chatbots have great potential to provide companions for the older adults to reduce their sense of loneliness [89]. For future researchers, the impact of GenAI chatbots on these two age groups is worth more investigation to ensure future more targeted usage.

As for settings, although a few studies implement AI chatbots in therapy procedures or educational settings, most studies have not yet streamlined GenAI chatbots in usual care procedures. Future research could investigate ways to integrate GenAI chatbots in existing treatment processes or programs to ensure sustainability of benefiting from AI chatbots. Apart from clinical settings, the application of GenAI chatbots in reducing anxiety and depression in educational settings is equally important, but only four studies investigated this area. Future studies could explore more diverse settings, including medical, educational, and therapy settings.

### Ethical Considerations

The growing use of social chatbots in mental health contexts raises significant ethical concerns that cannot be overlooked. News reports of suicide cases linked to interaction with AI companion chatbots [90,91] highlight the urgent risks, reinforcing findings from prior studies on the dark side of AI companionship, including emotional dependency, manipulation, privacy violations, and social isolation [92,93]. These dangers are particularly acute in mental health settings, where users may be especially vulnerable and the generative nature of AI systems can produce responses that are unpredictable, inappropriate, or even harmful.

Addressing these challenges requires concerted, multi-stakeholder efforts involving policymakers, technology developers, clinicians, and end-users. Robust regulatory frameworks, ethical guidelines, and oversight mechanisms are essential to ensure that generative AI chatbots are designed, deployed, and monitored in ways that safeguard user well-being [94,95]. This involves co-designing systems with input from mental health professionals and users; conducting systematic auditing and debiasing of training datasets; establishing safeguards to clearly delineate the boundaries of chatbot outputs; and ensuring that systems are regularly evaluated against therapeutic objectives [96,97]. Only through such comprehensive efforts can the potential benefits of GenAI chatbots be realized while minimizing risks to vulnerable populations.

### Limitation

Readers should be aware of a few limitations when interpreting the results. Among the 12 studies included in the meta-analysis, readers should be aware that some RCTs might have bias due to large baseline differences and differential attrition. First, some studies’ baseline differences exceed 0.25 SD, a threshold proposed by What Works Clearinghouse [98]. For example, Jeong [99] reported 0.30 SD for depression, McFadyen et al [61] reported 0.26 SD for anxiety, Sabour [64] reported 0.33 SD for depression. Second, some studies exceed 15% differential attrition between treatment and control groups, a threshold proposed by What Works Clearinghouse [98]. For instance, Chen et al [50] reported a differential attrition rate of 34% and He et al [54] reported 24 %. Third, the meta-analysis only analyzed a small sample of 12 studies. Although our number of effect sizes (n=37) is relatively large in terms of outcomes analyzed, it is small in terms of statistical analyses that combine empirical studies [100]. Readers should note that a small sample reduces statistical power for performing moderator analyses, and consequently, the capacity to obtain more precise estimates of the effect size via moderators [101]. Lastly, the risk of bias showed a mix of studies’ qualities between some concerns and high risk, which means that the GenAI mental health chatbot RCTs still need methodological improvement, and the results should be interpreted with caution.

### Conclusion

In conclusion, this systematic review has highlighted the promising yet inconsistent potential of GenAI chatbots in addressing mental health issues. Meta-regression findings indicate that social-oriented chatbots, as opposed to task-oriented ones, demonstrate greater effectiveness, though with wide variability and uncertainty. While these interventions are promising, their benefits come with risks that cannot be ignored. This review also identifies several research gaps, emphasizing the need for further investigation into adolescent and older adult populations, better serving users in non-WEIRD countries, analysis with mental health disorders other than anxiety and depression, integration of chatbots into existing therapy frameworks, and exploration within diverse settings. Given the substantial heterogeneity, moderate risk of bias, and small number of available RCTs, conclusions should be drawn with caution, viewing current findings as a foundation for more rigorous future studies rather than as definitive evidence of efficacy.

## Supplementary material

10.2196/78238 Multimedia Appendix 1 GenAI mental health chatbots’ comparison in features and target outcomes (organized by year of launch).

10.2196/78238 Multimedia Appendix 2 A comparison of the past nine systematic reviews on GenAI mental health chatbots.

10.2196/78238 Multimedia Appendix 3 Search keywords’ details for database searching.

10.2196/78238 Multimedia Appendix 4 Search string details recorded for database searching (Round 1: November 1, 2024; Round 2: March 5, 2025).

10.2196/78238 Multimedia Appendix 5 Univariate HKSJ-SJ meta-regression model results for models with and without moderators.

10.2196/78238 Multimedia Appendix 6 Statistical details of the univariate HKSJ model.

10.2196/78238 Checklist 1 PRISMA 2020 checklist.

## Abbreviations

AI: artificial intelligence
BERT: bidirectional encoder representations from transformers
CBT: cognitive behavioral therapy
df: degrees of freedom
GenAI: generative artificial intelligence
GPT: generative pre‑trained transformer
LLM: large language model
LSTM: long short-term memory
NA: not available
NLG: natural language generation
NLP: natural language processing
PI: prediction intervals
PRISMA: Preferred Reporting Items for Systematic Reviews and Meta-Analyses
RCT: randomized controlled trial
REML: restricted maximum likelihood
RoB2: revised Cochrane risk-of-bias tool for randomized trials
ROBINS-I: Risk Of Bias In Non-randomised Studies – of Interventions
SC: some concerns
SE: standard error
SMD: standardized mean difference
WEIRD: western, educated, industrialized, rich, and democratic
