# Supplementary material for: Generative AI Mental Health Chatbots as Therapeutic Tools: Systematic Review and Meta-Analysis of Their Role in Reducing Mental Health Issues
Source: J Med Internet Res. 2025 Dec 16;27:e78238. doi: 10.2196/78238 (PMC12707440; doi:10.2196/78238)
Supplement: Multimedia Appendix 1 [file jmir-v27-e78238-s001.docx]

**Supplementary Materials**

**Table A. GenAI Mental Health Chatbots’ Comparison in Features and Target Outcomes (Organized by Year of Launch)**

| **GenAI Mental Health Chatbot Names** | **License** | **Year of Launch** | **Country** | **Building Principles** | **Target Mental Health Outcomes** | **Publication** |
| --- | --- | --- | --- | --- | --- | --- |
| Help4Mood | Not yet commercialized | 2011 | European Union (UK, Spain, Romania, Italy) | CBT | Depression | [1] Burton, 2015 |
| imbPST | Not yet commercialized | 2016 | US | CBT | Depression | [2] Sandoval et al., 2016 |
| Tess | Free | 2016 | US | CBT | Depression, Anxiety | [3] Klos et al., 2021 |
| VIMSE (VR) | Not yet commercialized | 2016 | US | CBT | Spider Phobia, Depression, Anxiety | [4] Miloff et al., 2019 |
| Woebot | Free but need an access code | 2017 | US | CBT, IPT-A, DBT | Depression, Anxiety | [5] Fitzpatrick et al., 2017 |
| Vivibot | Free | 2018 | US | PPI | Depression, Anxiety | [6] Greer et al., 2019 |
| Xploro | Free | 2020 | UK | IP | Anxiety | [7] Bray, 2020 |
| Elomia | Elomia Pro 3 Months $48.99 | 2020 | Ukraine | CBT | Anxiety, Depression | [8] Romanovskyi et al., 2021 |
| Todaki | Not yet commercialized | 2020 | South Korea | CBT | Panic and Phobia, Anxiety and Depression | [9] Oh et al., 2020 |
| Zemedy | £49.99 / 3 months (Program only)  £169.99 / 3 months (Program + coaching) | 2021 | US | CBT | Depression, Anxiety | [10] Hunt et al., 2021 |
| Woebot-SUDs | Free but need an access code | 2021 | US | CBT | Depression, Anxiety, and Pandemic-related Mental Health Effects | [11] Prochaska, 2021 |
| XiaoNan | Not yet commercialized | 2022 | China | CBT | Depression, Anxiety | [12] Liu et al., 2022 |
| XiaoE | Not yet commercialized | 2022 | China | CBT | Depression | [13] He et al., 2022 |
| Fido | Not yet commercialized | 2022 | Polish | CBT | Depressive, Anxiety | [14] Karkosz et al., 2024 |
| Minder | Not yet commercialized | 2024 | Canada | Not Reported | Depression, Anxiety | [15] Nicol et al., 2022 |
| *Note. Woebot-SUDs = Woebot for Substance Use Disorder; CBT = Cognitive Behavioral Therapy; DBT = Dialectical Behavior Therapy; IP = Information Pull; IPT-A = Interpersonal Psychotherapy for Adolescents; PPI = Positive psychology–based intervention* | | | | | | |

**References**

[1] Burton C, Szentagotai Tatar A, McKinstry B, et al. Pilot randomised controlled trial of Help4Mood, an embodied virtual agent-based system to support treatment of depression. *J Telemed Telecare*. 2016;22(6):348-355. doi:10.1177/1357633X15609793

[2] Sandoval LR, Buckey JC, Ainslie R, Tombari M, Stone W, Hegel MT. Randomized Controlled Trial of a Computerized Interactive Media-Based Problem Solving Treatment for Depression. *Behav Ther*. 2017;48(3):413-425. doi:10.1016/j.beth.2016.04.001

[3] Klos MC, Escoredo M, Joerin A, Lemos VN, Rauws M, Bunge EL. Artificial Intelligence-Based Chatbot for Anxiety and Depression in University Students: Pilot Randomized Controlled Trial. *JMIR Form Res*. 2021;5(8):e20678. Published 2021 Aug 12. doi:10.2196/20678

[4] Miloff A, Lindner P, Dafgård P, et al. Automated virtual reality exposure therapy for spider phobia vs. in-vivo one-session treatment: A randomized non-inferiority trial. *Behav Res Ther*. 2019;118:130-140. doi:10.1016/j.brat.2019.04.004

[5] Fitzpatrick KK, Darcy A, Vierhile M. Delivering Cognitive Behavior Therapy to Young Adults With Symptoms of Depression and Anxiety Using a Fully Automated Conversational Agent (Woebot): A Randomized Controlled Trial. *JMIR Ment Health*. 2017;4(2):e19. Published 2017 Jun 6. doi:10.2196/mental.7785

[6] Greer S, Ramo D, Chang YJ, Fu M, Moskowitz J, Haritatos J. Use of the Chatbot "Vivibot" to Deliver Positive Psychology Skills and Promote Well-Being Among Young People After Cancer Treatment: Randomized Controlled Feasibility Trial. *JMIR Mhealth Uhealth*. 2019;7(10):e15018. Published 2019 Oct 31. doi:10.2196/15018

[7] Sabour S, Zhang W, Xiao X, et al. A chatbot for mental health support: exploring the impact of Emohaa on reducing mental distress in China. *Front Digit Health*. 2023;5:1133987. Published 2023 May 4. doi:10.3389/fdgth.2023.1133987

[8] Romanovskyi O, Pidbutska N, Knysh A. Elomia Chatbot: The effectiveness of artificial intelligence in the fight for mental health. *Proceedings of the International Conference on Computational Linguistics and Intelligent Systems (COLINS 2021)*; 2021. Available from:<https://api.semanticscholar.org/CorpusID:235271819>

[9] Oh J, Jang S, Kim H, Kim JJ. Efficacy of mobile app-based interactive cognitive behavioral therapy using a chatbot for panic disorder. *Int J Med Inform*. 2020;140:104171. doi:10.1016/j.ijmedinf.2020.104171

[10] Hunt M, Miguez S, Dukas B, Onwude O, White S. Efficacy of Zemedy, a Mobile Digital Therapeutic for the Self-management of Irritable Bowel Syndrome: Crossover Randomized Controlled Trial. *JMIR Mhealth Uhealth*. 2021;9(5):e26152. Published 2021 May 20. doi:10.2196/26152

[11] Prochaska JJ, Vogel EA, Chieng A, et al. A randomized controlled trial of a therapeutic relational agent for reducing substance misuse during the COVID-19 pandemic. *Drug Alcohol Depend*. 2021;227:108986. doi:10.1016/j.drugalcdep.2021.108986

[12] Liu H, Peng H, Song X, Xu C, Zhang M. Using AI chatbots to provide self-help depression interventions for university students: A randomized trial of effectiveness. *Internet Interv*. 2022;27:100495. Published 2022 Jan 6. doi:10.1016/j.invent.2022.100495

[13] He Y, Yang L, Zhu X, et al. Mental Health Chatbot for Young Adults With Depressive Symptoms During the COVID-19 Pandemic: Single-Blind, Three-Arm Randomized Controlled Trial. *J Med Internet Res*. 2022;24(11):e40719. Published 2022 Nov 21. doi:10.2196/40719

[14] Karkosz S, Szymański R, Sanna K, Michałowski J. Effectiveness of a Web-based and Mobile Therapy Chatbot on Anxiety and Depressive Symptoms in Subclinical Young Adults: Randomized Controlled Trial. *JMIR Form Res*. 2024;8:e47960. Published 2024 Mar 20. doi:10.2196/47960

[15] Nicol G, Wang R, Graham S, Dodd S, Garbutt J. Chatbot-Delivered Cognitive Behavioral Therapy in Adolescents With Depression and Anxiety During the COVID-19 Pandemic: Feasibility and Acceptability Study. *JMIR Form Res*. 2022;6(11):e40242. Published 2022 Nov 22. doi:10.2196/40242
