# Supplementary material for: Generative AI Mental Health Chatbots as Therapeutic Tools: Systematic Review and Meta-Analysis of Their Role in Reducing Mental Health Issues
Source: J Med Internet Res. 2025 Dec 16;27:e78238. doi: 10.2196/78238 (PMC12707440; doi:10.2196/78238)
Supplement: Multimedia Appendix 2 [file jmir-v27-e78238-s002.docx]

**Supplementary Materials**

**Table B. A Comparison of Past Nine Systematic Reviews on GenAI Mental Health Chatbots**

| **Citation** | **Journal** | **Participants** | **Method** | **Included studies** | **Finding (effect size)** | **Target chatbot** | **Target outcome** | **Search duration** | **Geographic Scope** | **Study Design** |
| --- | --- | --- | --- | --- | --- | --- | --- | --- | --- | --- |
| Gaffney et al (2019) [1] | JMIR mental health | M_age_=16-75 | Systematic review | 13 | All included studies reported reduced psychological distress postintervention with a conversational agent. In addition, 5 controlled studies demonstrated significant reductions in psychological distress compared with an information or no treatment control group with small-to-large effects. | Autonomous conversational agent | Depression, anxiety, specific phobia (heights), loneliness, and psychological distress | Up to January 2019 | United Kingdom, United States, Sweden, Japan | Quantitative Studies |
| Vaidyam et al (2019) [2] | Canadian Journal of psychiatry | M_age_=21.1-46.5 | Systematic review | 10 | For a few that examined the effectiveness, the conversational agent reduce depressive symptoms and detect their depressive symptoms | Conversational agent | Depression, anxiety, schizophrenia, bipolar, and substance abuse disorders. | Up to June 2018 | Various global studies | Quantitative Studies |
| Omarov et al (2023) [3] | Computers, Materials and Continua | n/a | Systematic review | 39 | Reduced anxiety and depression; increased user engagement. Despite access to medical conversational agents, many hesitate to adopt AI solutions. Engineers should apply theory-based techniques to address concerns and provide ethical, effective services to close healthcare gaps. | Artificial intelligence-enabled chatbot | Depression, anxiety, panic disorder, suicidal thinking, mood disorders, etc. | January 2017 - June 2021 | Various global studies | Quantitative Studies |
| Bhatt et al (2024) [4] | Annals of Neurosciences | Sample varies | Systematic review | 13 | The results obtained showed the significant positive consequences of using AI-based approaches in treating mental health issues | Majority of AI-based chatbot | Anxiety, depression, relationship issues, stress, OCD-related issues, persistent somatic symptom distress | 2018-May 2023 | Various global studies (Italy, Brazil, US, South Korea, India, New Zealand, Germany, Netherlands, Australia, Sweden and England) | RCT and quasi-experimental |
| He et al (2023) [5] | J Med Internet Res | N = 6089, M_age_= 36.32. | Systematic review and Meta-analysis | 32 RCTs were included in the systematic review and meta-analysis | Effect sizes (g=0.24–0.62) were robust, with long-term effects for depression, distress, and stress (g=0.16–0.39). Chatbots excelled in generalized anxiety; ECAs in depression and stress; VR CAs in specific anxiety; avatars in distress. Empathy improved outcomes, while increased interaction frequency enhanced efficacy. | Conversational agent interventions | Depressive symptoms; generalized anxiety symptoms; specific anxiety symptoms (phobia, social anxiety, panic); quality of life/well-being; general distress; stress; mental disorder symptoms (substance use, ADHD, psychosis); psychosomatic disease symptoms (chronic pain, IBS); positive and negative affect. | Up to May 1, 2022 | US, UK, Sweden; Japan, Korea, Switzerland, and Canada; Argentina, China, Ireland, the Netherlands, Germany, and New Zealand. | RCT |
| Abd-Alrazaq et al (2020) [6] | J Med Internet Res | M_age_= 31.3 | Systematic review and Meta-analysis | 35 in systematic review, 10^*^ RCTs in Meta-analysis | Decrease depression (g = -0.55), stress decrease (but high risk of bias), acrophobia decrease. Not significant for anxiety, psychological wellbeing. Conflicting for positive and negative affect; Safe to use chatbot | Chatbots | Effectiveness (e.g., severity or frequency of any mental disorders and psychological wellbeing) or safety (e.g., adverse events, deaths, admissions to psychiatric settings) of chatbots. | Up to June 11, 2019 | Global studies from multiple countries | RCT and quasi-experimental |
| Li et al (2023) [7] | npj Digital Medicine | N = 17,123 (15 countries/regions), M_age_=10.7 - 92 | Systematic review and meta-analysis | 35 in systematic review, 15 RCTs in meta-analysis | AI-based CAs significantly reduced depression (g=0.64, 95% CI 0.17–1.12) and distress (g=0.7, 95% CI 0.18–1.22), with no significant improvement in psychological well-being (g=0.32, 95% CI –0.13 to 0.78). Effects were stronger in multimodal, GenAI designs, mobile integration, and among clinical, subclinical, and elderly populations. | AI-based conversational agents | Depression, distress and psychological well-being | Up to May 26, 2023 | Global studies from multiple countries | RCT and quasi-experimental design |
| Zhong et al (2024) [8] | Journal of Affective Disorders | N = 3,477 | Systematic review and meta-analysis | 18 | Depression (g = -0.26, 95% CI -0.34 to -0.17) and anxiety (g = -0.19, 95% CI -0.29 to -0.09) improved, with the greatest benefits at 8 weeks but no significant effects at three-month follow-up | Mobile apps equipped with artificial intelligence-based chatbots | Depression and anxiety | Up to July 14, 2023 | Global studies from multiple countries | RCT |
| Villarreal-Zegarra et al (2024) [9] | JMIR Ment Health | NA | Systematic review and meta-analysis | 21 | Self-administered interventions based on NLP models were significantly more effective in reducing both depressive (SMD = 0.819, 95% CI 0.389-1.250; P<.001) and anxiety (SMD = 0.272, 95% CI 0.116-0.428; P=.001) symptoms compared to various control conditions | Interventions based on Natural language processing (NLP) | Depression and anxiety | Until November 3, 2023 | Global studies from multiple countries | RCT and quasi-experimental design |

***Note.*** The 10^*^ RCTs are categorized as follows: 4 focus on depression, 2 on anxiety, 2 on stress, and 2 on psychological distress. ADHD = attention-deficit/hyperactivity disorder; IBS = irritable bowel syndrome; US = The United States of America; UK = United Kingdom. Only systematic reviews of quantitative designs were included in this table. We excluded critical appraisal or qualitative systematic reviews or scoping reviews. We focused on AI chatbots, therefore, reviews that examine robots’ or social robots’ impact on mental health were excluded.

**References**

[1] Gaffney H, Mansell W, Tai S. Conversational agents in the treatment of mental health problems: Mixed-method systematic review. JMIR Ment Health. 2019 Oct 18;6(10):e14166.

[2] Vaidyam AN, Wisniewski H, Halamka JD, Kashavan MS, Torous JB. Chatbots and conversational agents in Mental Health: A review of the Psychiatric Landscape. The Canadian Journal of Psychiatry. 2019 Mar 21;64(7):456–64. doi:10.1177/0706743719828977

[3] Omarov B, Narynov S, Zhumanov Z. Artificial Intelligence-Enabled Chatbots in Mental Health: A Systematic Review. CMC. 2022;74(3):5105–22.

[4] Bhatt S. Digital Mental Health: Role of Artificial Intelligence in Psychotherapy. Annals of Neurosciences. 2024 Apr 22;32(2):117–27. doi:10.1177/09727531231221612

[5] He Y, Yang L, Qian C, Li T, Su Z, Zhang Q, et al. Conversational agent interventions for mental health problems: Systematic review and meta-analysis of randomized controlled trials. Journal of Medical Internet Research [Internet]. 2023 Apr 28 [cited 2025 Mar 16];25(1):e43862. Available from: https://www.jmir.org/2023/1/e43862

[6] Abd-Alrazaq AA, Alajlani M, Alalwan AA, Bewick BM, Gardner P, Househ M. An overview of the features of chatbots in mental health: A scoping review. Int J Med Inform. 2019 Dec;132:103978.

[7] Li H, Zhang R, Lee Y-C, Kraut RE, Mohr DC. Systematic Review and meta-analysis of AI-based conversational agents for promoting mental health and well-being. npj Digital Medicine. 2023 Dec 19;6(1). doi:10.1038/s41746-023-00979-5

[8] Zhong W, Luo J, Zhang H. The therapeutic effectiveness of artificial intelligence-based Chatbots in alleviation of depressive and anxiety symptoms in short-course treatments: A systematic review and meta-analysis. Journal of Affective Disorders. 2024 Jul;356:459–69. doi:10.1016/j.jad.2024.04.057

[9] Villarreal-Zegarra D, Reategui-Rivera CM, García-Serna J, Quispe-Callo G, Lázaro-Cruz G, Centeno-Terrazas G, et al. Self-Administered Interventions Based on Natural Language Processing Models for Reducing Depressive and Anxiety Symptoms: Systematic Review and Meta-Analysis. JMIR Mental Health. 2024 Aug 21;11(1):e59560.
