# Supplementary material for: Generative AI Mental Health Chatbots as Therapeutic Tools: Systematic Review and Meta-Analysis of Their Role in Reducing Mental Health Issues
Source: J Med Internet Res. 2025 Dec 16;27:e78238. doi: 10.2196/78238 (PMC12707440; doi:10.2196/78238)
Supplement: Multimedia Appendix 3 [file jmir-v27-e78238-s003.docx]

**Supplementary Materials**

**Table C. Search keywords’ details for database searching**

| **Method Keywords** | **Generative Keywords** | **Chatbot Keywords** | **Mental Health Keywords** | **Databases** | **Searched Date** |
| --- | --- | --- | --- | --- | --- |
| "intervention" OR "program" OR "workshop" OR "module" OR "course" | “OpenAI” OR “Large language model” OR “Generative*” OR “Elomia” OR “Replika” OR “XiaoE” OR “GPT-2” OR “GPT-3” OR “GPT-3.4” OR “GPT-4.5” OR “Gemini” OR “Co-pilot” OR “Perplexity” OR “LLaMA” OR “PaLM 1” OR “PaLM 2” OR “BERT” OR “LSTM” OR “NLP” OR “DP” OR “DALL·E 2” OR “Stable Diffusion” OR “AlphaCode” OR “neural-network” OR “Midjourney” OR “BLOOM” | "Chatbot*" OR "Artificial Intelligence" OR "Automated conversational agent" OR "Integrative psychological artificial intelligence" OR "automated smartphone-based chatbot" OR "Conversational agent in virtual reality" OR "Embodied conversational agent" OR "robot*" OR "social bot*" OR "dialogue system*" OR "conversational agent*" OR "conversational bot*" OR "conversational system*" OR "conversational interface*" OR "chatterbot*" OR "chatter bot*" OR "chat-bot*" OR "smartbot*" OR "smart bot*" OR "smart-bot*" OR "virtual coach*" OR "virtual agent*" OR "embodied agent*" OR "relational agent*" OR "avatar*" OR "virtual character*" OR "animated character*" OR "virtual human*" OR "virtual assistant*" OR "digital assistant*" OR "counseling agent*" OR "Artificial intelligence-empowered agents" | "mental illness" OR "mental disorder*" OR "suicid*" OR "affective disorder*" OR "psychotic disorder*" OR "post-traumatic stress disorder*" OR "PTSD" OR "distress" OR "depress*" OR "anxiety" OR "bipolar" OR "schizophrenia" OR "psychosis" OR "mental health" OR "negative affect*" OR "negative emotion*" | APA PsycArticles, APA PsycInfo, MEDLINE, OpenDissertations | 2014-2024 |

*Note. The search was completed using a Boolean Combination of Method AND Generative AND Chatbot AND Mental Health. Within each cluster, synonyms were combined by OR.*
