# Supplementary material for: Generative AI Mental Health Chatbots as Therapeutic Tools: Systematic Review and Meta-Analysis of Their Role in Reducing Mental Health Issues
Source: J Med Internet Res. 2025 Dec 16;27:e78238. doi: 10.2196/78238 (PMC12707440; doi:10.2196/78238)
Supplement: Multimedia Appendix 4 [file jmir-v27-e78238-s004.docx]

**Supplementary Materials**

**Section A. Search string details recorded for database searching** (Round 1: November 1^st^, 2024; Round 2: March 5^th^, 2025)

*All search was restricted to English language and 2014 to present if applicable.*

**Scopus:** TITLE ("OpenAI" or "Large language model" or "Generative*" or "Elomia" or "Replika" or "XiaoE" or "GPT-2" or "GPT-3" or "GPT-3.4" or "GPT-4.5" or "Gemini" or "Co-pilot" or "Perplexity" or "LLaMA" or "PaLM 1" or "PaLM 2" or "BERT" or "LSTM" or "NLP" or "DP" or "DALL·E 2" or "Stable Diffusion" or "AlphaCode" or "neural-network" or "Midjourney" or "BLOOM") and ("intervention" or "program" or "workshop" or "module" or "course") and ("Chatbot*" or "Artificial Intelligence" or "Automated conversational agent" or "Integrative psychological artificial intelligence" or "automated smartphone-based chatbot" or "Conversational agent in virtual reality" or "Embodied conversational agent" or "robot*" or "social bot*" or "dialogue system*" or "conversational agent*" or "conversational bot*" or "conversational system*" or "conversational interface*" or "chatterbot*" or "chatter bot*" or "chat-bot*" or "smartbot*" or "smart bot*" or "smart-bot*" or "virtual coach*" or "virtual agent*" or "embodied agent*" or "relational agent*" or "avatar*" or "virtual character*" or "animated character*" or "virtual human*" or "virtual assistant*" or "digital assistant*" or "counseling agent*" or "Artificial intelligence-empowered agents") and ("mental illness" or "mental disorder*" or "suicid*" or "affective disorder*" or "psychotic disorder*" or "post-traumatic stress disorder*" or "PTSD" or "distress" or "depress*" or "anxiety" or "bipolar" or "schizophrenia" or "psychosis" or "mental health" or "negative affect*" or "negative emotion*") AND ( LIMIT-TO ( LANGUAGE , "English" ) ) AND PUBYEAR > 2013 AND PUBYEAR < 2026

**MEDLINE:** AB ( ("OpenAI" or "Large language model" or "Generative*" or "Elomia" or "Replika" or "XiaoE" or "GPT-2" or "GPT-3" or "GPT-3.4" or "GPT-4.5" or "Gemini" or "Co-pilot" or "Perplexity" or "LLaMA" or "PaLM 1" or "PaLM 2" or "BERT" or "LSTM" or "NLP" or "DP" or "DALL·E 2" or "Stable Diffusion" or "AlphaCode" or "neural-network" or "Midjourney" or "BLOOM") and ("intervention" or "program" or "workshop" or "module" or "course") and ("Chatbot*" or "Artificial Intelligence" or "Automated conversational agent" or "Integrative psychological artificial intelligence" or "automated smartphone-based chatbot" or "Conversational agent in virtual reality" or "Embodied conversational agent" or "robot*" or "social bot*" or "dialogue system*" or "conversational agent*" or "conversational bot*" or "conversational system*" or "conversational interface*" or "chatterbot*" or "chatter bot*" or "chat-bot*" or "smartbot*" or "smart bot*" or "smart-bot*" or "virtual coach*" or "virtual agent*" or "embodied agent*" or "relational agent*" or "avatar*" or "virtual character*" or "animated character*" or "virtual human*" or "virtual assistant*" or "digital assistant*" or "counseling agent*" or "Artificial intelligence-empowered agents") and ("mental illness" or "mental disorder*" or "suicid*" or "affective disorder*" or "psychotic disorder*" or "post-traumatic stress disorder*" or "PTSD" or "distress" or "depress*" or "anxiety" or "bipolar" or "schizophrenia" or "psychosis" or "mental health" or "negative affect*" or "negative emotion*")).mp. [mp=title, book title, abstract, original title, name of substance word, subject heading word, floating sub-heading word, keyword heading word, organism supplementary concept word, protocol supplementary concept word, rare disease supplementary concept word, unique identifier, synonyms, population supplementary concept word, anatomy supplementary concept word]) limit 2 to (english language and yr = “2014-Current”)

**EMBASE:** (("OpenAI" or "Large language model" or "Generative*" or "Elomia" or "Replika" or "XiaoE" or "GPT-2" or "GPT-3" or "GPT-3.4" or "GPT-4.5" or "Gemini" or "Co-pilot" or "Perplexity" or "LLaMA" or "PaLM 1" or "PaLM 2" or "BERT" or "LSTM" or "NLP" or "DP" or "DALL·E 2" or "Stable Diffusion" or "AlphaCode" or "neural-network" or "Midjourney" or "BLOOM") and ("intervention" or "program" or "workshop" or "module" or "course") and ("Chatbot*" or "Artificial Intelligence" or "Automated conversational agent" or "Integrative psychological artificial intelligence" or "automated smartphone-based chatbot" or "Conversational agent in virtual reality" or "Embodied conversational agent" or "robot*" or "social bot*" or "dialogue system*" or "conversational agent*" or "conversational bot*" or "conversational system*" or "conversational interface*" or "chatterbot*" or "chatter bot*" or "chat-bot*" or "smartbot*" or "smart bot*" or "smart-bot*" or "virtual coach*" or "virtual agent*" or "embodied agent*" or "relational agent*" or "avatar*" or "virtual character*" or "animated character*" or "virtual human*" or "virtual assistant*" or "digital assistant*" or "counseling agent*" or "Artificial intelligence-empowered agents") and ("mental illness" or "mental disorder*" or "suicid*" or "affective disorder*" or "psychotic disorder*" or "post-traumatic stress disorder*" or "PTSD" or "distress" or "depress*" or "anxiety" or "bipolar" or "schizophrenia" or "psychosis" or "mental health" or "negative affect*" or "negative emotion*")).ti,ab.

**CINAHL:** AB (("OpenAI" or "Large language model" or "Generative*" or "Elomia" or "Replika" or "XiaoE" or "GPT-2" or "GPT-3" or "GPT-3.4" or "GPT-4.5" or "Gemini" or "Co-pilot" or "Perplexity" or "LLaMA" or "PaLM 1" or "PaLM 2" or "BERT" or "LSTM" or "NLP" or "DP" or "DALL·E 2" or "Stable Diffusion" or "AlphaCode" or "neural-network" or "Midjourney" or "BLOOM") and ("intervention" or "program" or "workshop" or "module" or "course") and ("Chatbot*" or "Artificial Intelligence" or "Automated conversational agent" or "Integrative psychological artificial intelligence" or "automated smartphone-based chatbot" or "Conversational agent in virtual reality" or "Embodied conversational agent" or "robot*" or "social bot*" or "dialogue system*" or "conversational agent*" or "conversational bot*" or "conversational system*" or "conversational interface*" or "chatterbot*" or "chatter bot*" or "chat-bot*" or "smartbot*" or "smart bot*" or "smart-bot*" or "virtual coach*" or "virtual agent*" or "embodied agent*" or "relational agent*" or "avatar*" or "virtual character*" or "animated character*" or "virtual human*" or "virtual assistant*" or "digital assistant*" or "counseling agent*" or "Artificial intelligence-empowered agents") and ("mental illness" or "mental disorder*" or "suicid*" or "affective disorder*" or "psychotic disorder*" or "post-traumatic stress disorder*" or "PTSD" or "distress" or "depress*" or "anxiety" or "bipolar" or "schizophrenia" or "psychosis" or "mental health" or "negative affect*" or "negative emotion*"))

**Web of Science:** AB=(("OpenAI" or "Large language model" or "Generative*" or "Elomia" or "Replika" or "XiaoE" or "GPT-2" or "GPT-3" or "GPT-3.4" or "GPT-4.5" or "Gemini" or "Co-pilot" or "Perplexity" or "LLaMA" or "PaLM 1" or "PaLM 2" or "BERT" or "LSTM" or "NLP" or "DP" or "DALL·E 2" or "Stable Diffusion" or "AlphaCode" or "neural-network" or "Midjourney" or "BLOOM") and ("intervention" or "program" or "workshop" or "module" or "course") and ("Chatbot*" or "Artificial Intelligence" or "Automated conversational agent" or "Integrative psychological artificial intelligence" or "automated smartphone-based chatbot" or "Conversational agent in virtual reality" or "Embodied conversational agent" or "robot*" or "social bot*" or "dialogue system*" or "conversational agent*" or "conversational bot*" or "conversational system*" or "conversational interface*" or "chatterbot*" or "chatter bot*" or "chat-bot*" or "smartbot*" or "smart bot*" or "smart-bot*" or "virtual coach*" or "virtual agent*" or "embodied agent*" or "relational agent*" or "avatar*" or "virtual character*" or "animated character*" or "virtual human*" or "virtual assistant*" or "digital assistant*" or "counseling agent*" or "Artificial intelligence-empowered agents") and ("mental illness" or "mental disorder*" or "suicid*" or "affective disorder*" or "psychotic disorder*" or "post-traumatic stress disorder*" or "PTSD" or "distress" or "depress*" or "anxiety" or "bipolar" or "schizophrenia" or "psychosis" or "mental health" or "negative affect*" or "negative emotion*"))

**ACM Digital Library:** ("OpenAI" or "Large language model" or "Generative*" or "Elomia" or "Replika" or "XiaoE" or "GPT-2" or "GPT-3" or "GPT-3.4" or "GPT-4.5" or "Gemini" or "Co-pilot" or "Perplexity" or "LLaMA" or "PaLM 1" or "PaLM 2" or "BERT" or "LSTM" or "NLP" or "DP" or "DALL·E 2" or "Stable Diffusion" or "AlphaCode" or "neural-network" or "Midjourney" or "BLOOM") and ("intervention" or "program" or "workshop" or "module" or "course") and ("Chatbot*" or "Artificial Intelligence" or "Automated conversational agent" or "Integrative psychological artificial intelligence" or "automated smartphone-based chatbot" or "Conversational agent in virtual reality" or "Embodied conversational agent" or "robot*" or "social bot*" or "dialogue system*" or "conversational agent*" or "conversational bot*" or "conversational system*" or "conversational interface*" or "chatterbot*" or "chatter bot*" or "chat-bot*" or "smartbot*" or "smart bot*" or "smart-bot*" or "virtual coach*" or "virtual agent*" or "embodied agent*" or "relational agent*" or "avatar*" or "virtual character*" or "animated character*" or "virtual human*" or "virtual assistant*" or "digital assistant*" or "counseling agent*" or "Artificial intelligence-empowered agents") and ("mental illness" or "mental disorder*" or "suicid*" or "affective disorder*" or "psychotic disorder*" or "post-traumatic stress disorder*" or "PTSD" or "distress" or "depress*" or "anxiety" or "bipolar" or "schizophrenia" or "psychosis" or "mental health" or "negative affect*" or "negative emotion*")

**ProQuest Dissertations & Theses Global:** ab ("OpenAI" or "Large language model" or "Generative*" or "Elomia" or "Replika" or "XiaoE" or "GPT-2" or "GPT-3" or "GPT-3.4" or "GPT-4.5" or "Gemini" or "Co-pilot" or "Perplexity" or "LLaMA" or "PaLM 1" or "PaLM 2" or "BERT" or "LSTM" or "NLP" or "DP" or "DALL·E 2" or "Stable Diffusion" or "AlphaCode" or "neural-network" or "Midjourney" or "BLOOM") and ("intervention" or "program" or "workshop" or "module" or "course") and ("Chatbot*" or "Artificial Intelligence" or "Automated conversational agent" or "Integrative psychological artificial intelligence" or "automated smartphone-based chatbot" or "Conversational agent in virtual reality" or "Embodied conversational agent" or "robot*" or "social bot*" or "dialogue system*" or "conversational agent*" or "conversational bot*" or "conversational system*" or "conversational interface*" or "chatterbot*" or "chatter bot*" or "chat-bot*" or "smartbot*" or "smart bot*" or "smart-bot*" or "virtual coach*" or "virtual agent*" or "embodied agent*" or "relational agent*" or "avatar*" or "virtual character*" or "animated character*" or "virtual human*" or "virtual assistant*" or "digital assistant*" or "counseling agent*" or "Artificial intelligence-empowered agents") and ("mental illness" or "mental disorder*" or "suicid*" or "affective disorder*" or "psychotic disorder*" or "post-traumatic stress disorder*" or "PTSD" or "distress" or "depress*" or "anxiety" or "bipolar" or "schizophrenia" or "psychosis" or "mental health" or "negative affect*" or "negative emotion*")

**PsycInfo:** AB ("OpenAI" or "Large language model" or "Generative*" or "Elomia" or "Replika" or "XiaoE" or "GPT-2" or "GPT-3" or "GPT-3.4" or "GPT-4.5" or "Gemini" or "Co-pilot" or "Perplexity" or "LLaMA" or "PaLM 1" or "PaLM 2" or "BERT" or "LSTM" or "NLP" or "DP" or "DALL·E 2" or "Stable Diffusion" or "AlphaCode" or "neural-network" or "Midjourney" or "BLOOM") and ("intervention" or "program" or "workshop" or "module" or "course") and ("Chatbot*" or "Artificial Intelligence" or "Automated conversational agent" or "Integrative psychological artificial intelligence" or "automated smartphone-based chatbot" or "Conversational agent in virtual reality" or "Embodied conversational agent" or "robot*" or "social bot*" or "dialogue system*" or "conversational agent*" or "conversational bot*" or "conversational system*" or "conversational interface*" or "chatterbot*" or "chatter bot*" or "chat-bot*" or "smartbot*" or "smart bot*" or "smart-bot*" or "virtual coach*" or "virtual agent*" or "embodied agent*" or "relational agent*" or "avatar*" or "virtual character*" or "animated character*" or "virtual human*" or "virtual assistant*" or "digital assistant*" or "counseling agent*" or "Artificial intelligence-empowered agents") and ("mental illness" or "mental disorder*" or "suicid*" or "affective disorder*" or "psychotic disorder*" or "post-traumatic stress disorder*" or "PTSD" or "distress" or "depress*" or "anxiety" or "bipolar" or "schizophrenia" or "psychosis" or "mental health" or "negative affect*" or "negative emotion*")

**Child Development & Adolescent Studies:** ("OpenAI" or "Large language model" or "Generative*" or "Elomia" or "Replika" or "XiaoE" or "GPT-2" or "GPT-3" or "GPT-3.4" or "GPT-4.5" or "Gemini" or "Co-pilot" or "Perplexity" or "LLaMA" or "PaLM 1" or "PaLM 2" or "BERT" or "LSTM" or "NLP" or "DP" or "DALL·E 2" or "Stable Diffusion" or "AlphaCode" or "neural-network" or "Midjourney" or "BLOOM") and ("intervention" or "program" or "workshop" or "module" or "course") and ("Chatbot*" or "Artificial Intelligence" or "Automated conversational agent" or "Integrative psychological artificial intelligence" or "automated smartphone-based chatbot" or "Conversational agent in virtual reality" or "Embodied conversational agent" or "robot*" or "social bot*" or "dialogue system*" or "conversational agent*" or "conversational bot*" or "conversational system*" or "conversational interface*" or "chatterbot*" or "chatter bot*" or "chat-bot*" or "smartbot*" or "smart bot*" or "smart-bot*" or "virtual coach*" or "virtual agent*" or "embodied agent*" or "relational agent*" or "avatar*" or "virtual character*" or "animated character*" or "virtual human*" or "virtual assistant*" or "digital assistant*" or "counseling agent*" or "Artificial intelligence-empowered agents") and ("mental illness" or "mental disorder*" or "suicid*" or "affective disorder*" or "psychotic disorder*" or "post-traumatic stress disorder*" or "PTSD" or "distress" or "depress*" or "anxiety" or "bipolar" or "schizophrenia" or "psychosis" or "mental health" or "negative affect*" or "negative emotion*")

**ERIC:** ("OpenAI" or "Large language model" or "Generative*" or "Elomia" or "Replika" or "XiaoE" or "GPT-2" or "GPT-3" or "GPT-3.4" or "GPT-4.5" or "Gemini" or "Co-pilot" or "Perplexity" or "LLaMA" or "PaLM 1" or "PaLM 2" or "BERT" or "LSTM" or "NLP" or "DP" or "DALL·E 2" or "Stable Diffusion" or "AlphaCode" or "neural-network" or "Midjourney" or "BLOOM") and ("intervention" or "program" or "workshop" or "module" or "course") and ("Chatbot*" or "Artificial Intelligence" or "Automated conversational agent" or "Integrative psychological artificial intelligence" or "automated smartphone-based chatbot" or "Conversational agent in virtual reality" or "Embodied conversational agent" or "robot*" or "social bot*" or "dialogue system*" or "conversational agent*" or "conversational bot*" or "conversational system*" or "conversational interface*" or "chatterbot*" or "chatter bot*" or "chat-bot*" or "smartbot*" or "smart bot*" or "smart-bot*" or "virtual coach*" or "virtual agent*" or "embodied agent*" or "relational agent*" or "avatar*" or "virtual character*" or "animated character*" or "virtual human*" or "virtual assistant*" or "digital assistant*" or "counseling agent*" or "Artificial intelligence-empowered agents") and ("mental illness" or "mental disorder*" or "suicid*" or "affective disorder*" or "psychotic disorder*" or "post-traumatic stress disorder*" or "PTSD" or "distress" or "depress*" or "anxiety" or "bipolar" or "schizophrenia" or "psychosis" or "mental health" or "negative affect*" or "negative emotion*")

**PsyArXiv:** title-abstract: ("OpenAI" or "Large language model" or "Generative*" or "Elomia" or "Replika" or "XiaoE" or "GPT-2" or "GPT-3" or "GPT-3.4" or "GPT-4.5" or "Gemini" or "Co-pilot" or "Perplexity" or "LLaMA" or "PaLM 1" or "PaLM 2" or "BERT" or "LSTM" or "NLP" or "DP" or "DALL·E 2" or "Stable Diffusion" or "AlphaCode" or "neural-network" or "Midjourney" or "BLOOM") and ("intervention" or "program" or "workshop" or "module" or "course") and ("Chatbot*" or "Artificial Intelligence" or "Automated conversational agent" or "Integrative psychological artificial intelligence" or "automated smartphone-based chatbot" or "Conversational agent in virtual reality" or "Embodied conversational agent" or "robot*" or "social bot*" or "dialogue system*" or "conversational agent*" or "conversational bot*" or "conversational system*" or "conversational interface*" or "chatterbot*" or "chatter bot*" or "chat-bot*" or "smartbot*" or "smart bot*" or "smart-bot*" or "virtual coach*" or "virtual agent*" or "embodied agent*" or "relational agent*" or "avatar*" or "virtual character*" or "animated character*" or "virtual human*" or "virtual assistant*" or "digital assistant*" or "counseling agent*" or "Artificial intelligence-empowered agents") and ("mental illness" or "mental disorder*" or "suicid*" or "affective disorder*" or "psychotic disorder*" or "post-traumatic stress disorder*" or "PTSD" or "distress" or "depress*" or "anxiety" or "bipolar" or "schizophrenia" or "psychosis" or "mental health" or "negative affect*" or "negative emotion*")
