# Supplementary material for: Generative AI Mental Health Chatbots as Therapeutic Tools: Systematic Review and Meta-Analysis of Their Role in Reducing Mental Health Issues
Source: J Med Internet Res. 2025 Dec 16;27:e78238. doi: 10.2196/78238 (PMC12707440; doi:10.2196/78238)
Supplement: Multimedia Appendix 5 [file jmir-v27-e78238-s005.docx]

**Supplementary Materials**

**Table E. Univariate HKSJ-SJ meta-regression model results for models with and without moderators.**

| **Coefficient** | **Comparison Group** | **SMD** | **SE** | **t** | **df** | **P Value** | ***95%PI*** |
| --- | --- | --- | --- | --- | --- | --- | --- |
| **Null Model for All Outcomes** | | | | | | |  |
| Intercept |  | 0.36 | 0.13 | 2.70 | 32 | 0.011* | -1.18, 1.90 |
| **Null Model for Depression** | | | | | | |  |
| Intercept |  | 0.49 | 0.21 | 2.39 | 7 | 0.048* | -0.90, 1.88 |
| **Null Model for Anxiety** | | | | | | |  |
| Intercept |  | 0.43 | 0.28 | 1.52 | 11 | 0.156 | -1.76, 2.61 |
| **Null Model for Negative Affect and Mood** | | | | | | |  |
| Intercept |  | 0.28 | 0.32 | 0.89 | 7 | 0.404 | -1.93, 2.50 |
| **Null Model for Stress** | | | | | | |  |
| Intercept |  | 0.10 | 0.05 | 1.90 | 3 | 0.153 | -0.12, 0.32 |
| **Single Predictor Model with Social Function** | | | | | | |  |
| Social Function: task-oriented | Social Function: social-oriented | -1.04 | 0.44 | 2.36 | 9.76 | 0.041* |  |
| **Single Predictor Model with Control Group** | |  |  |  |  |  |  |
| Control Group: Active | Control Group: Passive | -0.17 | 0.31 | -0.535 | 4.39 | 0.619 |  |
| **Single Predictor Model with Human Assistance** | |  |  |  |  |  |  |
| Human-assisted | Self-guided | -0.38 | 0.335 | -1.14 | 3.6 | 0.325 |  |

***Note.***  *SE = standard error; df = degrees of freedom; SMD = Standardized Mean Difference; PI = Predictive Intervals. * P < .05*
