# Supplementary material for: Generative AI Mental Health Chatbots as Therapeutic Tools: Systematic Review and Meta-Analysis of Their Role in Reducing Mental Health Issues
Source: J Med Internet Res. 2025 Dec 16;27:e78238. doi: 10.2196/78238 (PMC12707440; doi:10.2196/78238)
Supplement: Multimedia Appendix 6 [file jmir-v27-e78238-s006.docx]

**Supplementary Materials**

**Section B. Statistical details of the univariate HKSJ model.**

As a sensitivity analysis, we also tested intervention effects using the univariate HKSJ–SJ random-effects meta-analysis with 29 study-outcome pairs. As shown in Table 4, GenAI chatbot interventions can significantly reduce mental health issues (*ES* = 0.42, *p* = .008, *SE* = 0.15, N_ES_= 29, 95%PI = [-1.19, 2.04], 95% CI = [0.122, 0.774]). While the average effect is 0.42, the true effect in a new but similar population or setting is likely to range from -1.19 to 2.04. The 95% PIs suggest considerable between-study variability: in some real-world settings AI mental health chatbots may have negligible or even unfavorable effects, whereas in others the benefit may be moderate. The confidence interval means that we are 95% certain that the true average effect lies between 0.122 and 0.774. Since the confidence interval does not contain 0, we are more confident that GenAI chatbot interventions can significantly reduce mental health issues. There is a substantial amount of between-study heterogeneity [τ^2^ = 0.641; I^2^ = 95.3%, *Q* = 298.39, *p* < .001].
